# Blockchain-Based Authentication in Internet of Vehicles: A Survey

**DOI:** 10.3390/s21237927

**Published:** 2021-11-27

**Authors:** Sohail Abbas, Manar Abu Talib, Afaf Ahmed, Faheem Khan, Shabir Ahmad, Do-Hyeun Kim

**Affiliations:** 1 Department of Computer Science, College of Computing and Informatics, University of Sharjah, Sharjah 27272, United Arab Emirates; sabbas@sharjah.ac.ae (S.A.); mtalib@sharjah.ac.ae (M.A.T.); 2 College of Engineering, Al Ain University, Al Ain 64141, United Arab Emirates; afaf.ahmed@aau.ac.ae; 3 Department of Computer Engineering, Gachon University, Seongnam-si 13120, Korea; faheem@gachon.ac.kr; 4 Department of IT Convergence Engineering, Gachon University, Seongnam-si 13120, Korea; shabir@gachon.ac.kr; 5 Department of Computer Engineering, Jeju National University, Jeju 63243, Korea

**Keywords:** authentication, blockchain, Internet of Vehicles, Vehicular Ad-hoc Networks

## Abstract

Internet of Vehicles (IoV) has emerged as an advancement over the traditional Vehicular Ad-hoc Networks (VANETs) towards achieving a more efficient intelligent transportation system that is capable of providing various intelligent services and supporting different applications for the drivers and passengers on roads. In order for the IoV and VANETs environments to be able to offer such beneficial road services, huge amounts of data are generated and exchanged among the different communicated entities in these vehicular networks wirelessly via open channels, which could attract the adversaries and threaten the network with several possible types of security attacks. In this survey, we target the authentication part of the security system while highlighting the efficiency of blockchains in the IoV and VANETs environments. First, a detailed background on IoV and blockchain is provided, followed by a wide range of security requirements, challenges, and possible attacks in vehicular networks. Then, a more focused review is provided on the recent blockchain-based authentication schemes in IoV and VANETs with a detailed comparative study in terms of techniques used, network models, evaluation tools, and attacks counteracted. Lastly, some future challenges for IoV security are discussed that are necessary to be addressed in the upcoming research.

## 1. Introduction

With the huge increase in the number of vehicles on roads nowadays, more accidents and traffic congestion issues are encountered. This raises the need for serious arrangements to ensure roads’ safety and traffic efficiency. Different technologies have been introduced towards maintaining safer and time-efficient driving on roads, such as, Vehicular Ad-hoc Networks (VANETs) in which the vehicles exchange data about their speed, location, etc., and other road-related information to raise their awareness about surrounding road conditions and help them making better and effective decisions. However, with the rapid advancement in today’s technologies such as ubiquitous connectivity, wireless technologies, sensor devices, smart vehicles, and cloud computing platforms, the need for more powerful vehicular networks has increased. Hence, the IoV has appeared that can exploit and incorporate all these advanced technologies in order to provide more satisfying real-time services for vehicles’ drivers and passengers.

IoV has emerged with great potential to support various services and offer several benefits to the transportation system such as cost effectiveness, time efficiency, road safety [1], traffic management [2,3,4], evolution of smart cities [5,6], autonomous driving [7,8,9,10] alarms and dynamic warning systems [11,12,13] as well as recording fatal occurrences [14]. However, in order for the IoV system to be able to secure such services, enormous amounts of data need to be generated and exchanged among the different IoV entities including vehicles, pedestrians, and roadside infrastructure. Since this information exchange takes place through an open-channel wireless network, the exchanged messages are vulnerable to various security attacks that could undermine the privacy of the communicating entities and the confidentiality of their data via eavesdropping or even affect the integrity of the transmitted messages by tampering them before reaching their target destination. Other types of security attacks that could be encountered in IoV environments are the attacks that target the authenticity of users. Here, a malicious entity masquerades a legitimate user and may commit malicious activities in the network. Therefore, efficient authentication is necessary to prevent such attacks in IoV.

On the other hand, blockchain technology has recently drawn the attention of both industry and academia due to its efficient characteristics represented in decentralization, immutability, consensus, fault tolerance, and enhanced security. Blockchain was first known as the enabling technology behind Bitcoin or cryptocurrency. Yet, it has recently attracted various emerging domains such as smart cities [15,16,17], smart grids [18,19,20], Internet of Things (IoT) [21,22], Cyber Physical Systems (CPS) [23,24,25], robotics [26,27], machine learning [28,29], and health systems [30,31,32]. IoV platforms have also started to adopt blockchain for various services which include data management [33,34], resource trading [35,36], resource sharing [37,38], vehicle management [39,40], ride sharing [41,42], traffic control [43,44], and forensics applications [45,46]. In this paper, we highlight the use of blockchain in IoV and VANETs for authentication by surveying a number of recent blockchain-based authentication schemes.

Numerous surveys have recently been published discussing the authentication approaches and protocols in the vehicular networks of VANETs and IoV which are summarized in Table 1. Some of the surveys have focused on authentication in IoV and/or VANETs as part of the Intelligent Transportation Systems (ITS) whereas others have exhibited wider perspective by discussing IoV authentication as a subsection of the Internet of Things (IoT) technology.

In order to highlight the contribution of this paper, a number of recent state-of-art surveys are summarized and compared in Table 1. In [47], the cryptographic-based authentication protocols have been discussed in a wide range of IoT environments, namely, IoV, Internet of Sensors (IoS), Internet of Energy (IoE), and Machine to Machine Communication (M2M). In [48], a range of authentication schemes that are based on cryptography, digital signature, and message verification in the context of VANETs have been presented. IoV authentication has been implicitly reviewed in [49] by introducing a multi-criteria classification for the authentication schemes in the IoT environment in general. A broad range of crypto-based authentication schemes in VANETs environments [50,51] and IoV networks [52] have also been reviewed. However, despite discussing the authentication protocols from different points of view and introducing diverse categorization criteria, all the above surveys have the common factor of reviewing the cryptographic-based authentication schemes in IoV or VANETs and none of them has reviewed the authentication schemes that are based on blockchains.

On the other hand, blockchain-based applications in IoV have been addressed by many surveys recently. For instance, the authors in [53] have surveyed a number of blockchain-based applications that aim to improve the security, privacy, trust and cooperation in IoV networks. Seven different aspects where blockchain technology can be incorporated with IoV have been discussed in [54] such as IoV security, trust management, and data management. Moreover, different blockchain-based IoV security methods have been categorized and reviewed in [55]. Although these surveys might have mentioned a few blockchain-based authentication schemes in IoV, they have briefly mentioned them on the run as a small part of the broad field of IoV security and none of them has provided a detailed survey that is only dedicated to the blockchain-based authentication schemes in IoV. Thus, the main contributions of this survey are:

- Highlighting the significance of the blockchain technology in IoV and VANETs by presenting a wide range of the blockchain-based authentication schemes that are proposed in the recent literature.
- Providing the first detailed survey focusing on the application of blockchain technology to a specific aspect of IoV security; that is, the authentication. Considering both IoV and VANETs technologies when surveying the different blockchain-based authentication schemes instead of restricting them to only one vehicular technology, which can provide a comprehensive source for researchers interested in the field of blockchain-based authentication.

The organization of the paper is as follows: Section 2 presents some preliminary information related to IoV and blockchain. Then, the security requirements and challenges along with some common security attacks and threats that can be encountered in IoV and VANETs paradigms are discussed in Section 3. After that, the main part of the paper is represented by Section 4 and Section 5 where a wide range of blockchain-based authentication schemes in IoV and VANETs are reviewed and compared. Section 6 then suggests some future research challenges after which the paper is finally concluded in Section 7.

## 2. Background

### 2.1. Internet of Vehicles

IoV is an emerging field that mainly incorporates ITS and IoT technologies, while covering a wide range of other technologies and applications such as vehicular information services, advanced wireless communication technologies, cloud computing, edge computing, and automotive electronics to provide intelligent transportation services and enhance the quality of roads. It integrates the intelligent in-vehicle sensor devices with the intra-vehicle and inter-vehicle wireless communication technologies along with Internet technology to collect and exchange vehicle-related and traffic-related data that can be later used for making better road-related actions and decisions. IoV consists of three basic components: (1) the intra-vehicular network, (2) inter-vehicular network, and (3) vehicular mobile Internet [56]. This includes the communication between vehicles in the same vehicular network, the communication between different vehicular networks, and the connection between vehicles and mobiles, respectively. The functionality of IoV imposes equipping vehicles with several smart units and devices including electronic control units, On Board Units (OBUs), sensors, event data records, cameras, GPS modules as well as a diverse number of wired (Controller Area Network and Local Interconnect Network buses) and wireless (i.e., Bluetooth) communication technologies.

The former technology to IoV is the VANETS which was basically introduced to improve the traffic efficiency and road safety by establishing connectivity and exchanging information between the moving vehicles with and without the aid of any pre-established roadside infrastructure via different communication modes namely, Wireless Access in Vehicular Environments (WAVE) based Wi-Fi, ad-hoc, and hybrid. Despite its efficiency in addressing road safety and traffic management issues with low operational cost, VANETs exhibit some commercialization problems which include but are not limited to the following [57]:

1. VANETs’ framework could not fully support the global and sustainable services targeted by ITS applications. This is caused by the pure ad-hoc communication architecture, in which an on-road vehicle can lose its granted services once it disconnects from an ad-hoc network. This is due to the inability of collaborating with other alternative reachable networks.
2. Internet connectivity in VANETS could not be ensured, which affects the availability of commercial applications for vehicles’ drivers and passengers since those applications rely on reliable Internet connectivity.
3. Despite the rapid technological advancement of personal mobile devices, they could not communicate with VANETs due to the incompatible network architecture.
4. Intelligent decision making, and big data analytics applications were not possible in certain VANETs architectures. This could be related to the computing and storage constraints and the lack of cloud computing services.
5. The application services could not guarantee high level of accuracy, since VANETs localize the computation and processing of traffic data information.

The above limitations of VANETs have drawn the attention of researchers and industrial developers to extend the capabilities of the existing vehicular networks to move further steps towards providing more efficient vehicular services and achieving the global objectives of ITS. Consequently, IoV has emerged as an advanced vehicular technology that attempts to overcome the shortcomings of conventional VANETs through supporting a high range of mobility, strong connectivity among vehicles and with roadside infrastructure, reliable Internet connection, and high interactivity with personal devices. IoV can also provide an immediate management of risk situations through maintaining low delay and delivering high reliability and robustness. Cloud and edge computing capabilities, processing, and analysis of collected data to transform them into useful information through big data analytics tools to provide services to consumers and businesses are as well positive points for the IoV.

**Figure 1:**
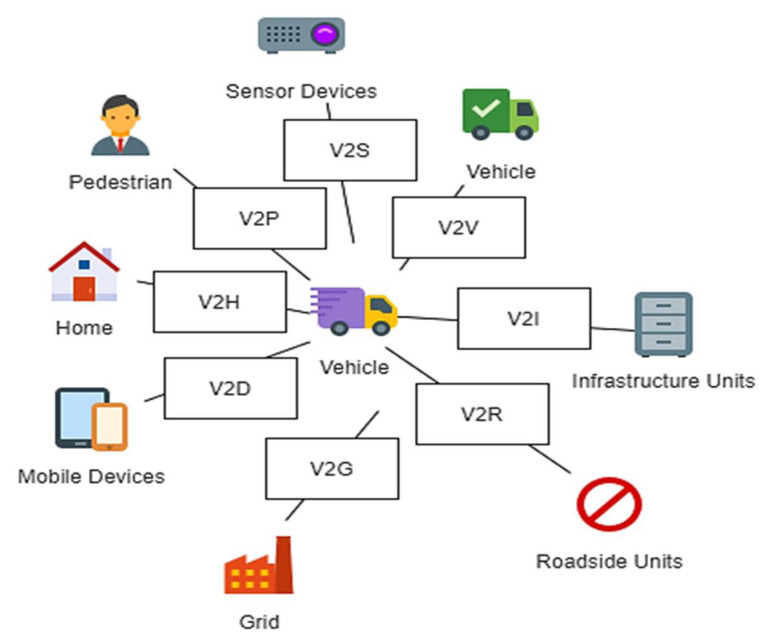
Communication models in IoV.

In addition, IoV has brought the ability to support diverse types of interaction models including Vehicle-to-Vehicle (V2V), Vehicle-to-Roadside-unit (V2R), Vehicle-to-Infrastructure (V2I), Vehicle-to-Pedestrian (V2P), Vehicle-to-Sensor (V2S), and many others as indicated in Figure 1. V2V and V2R indicate the interaction among the vehicles and the interaction between the vehicles and the RSUs, respectively, via wireless protocols such as WAVE. V2I is the communication between vehicles and infrastructure possibly via Wi-Fi, Long Term Evolution (LTE), or 5G. While V2S represents the onboard sensor communication via Ethernet and Wi-Fi, V2P refers to the communication among vehicles and personal devices such as smartphones via Apple’s CarPlay, Open Automotive Alliance Android system, or Near Field Communication [58,59].

**Figure 2:**
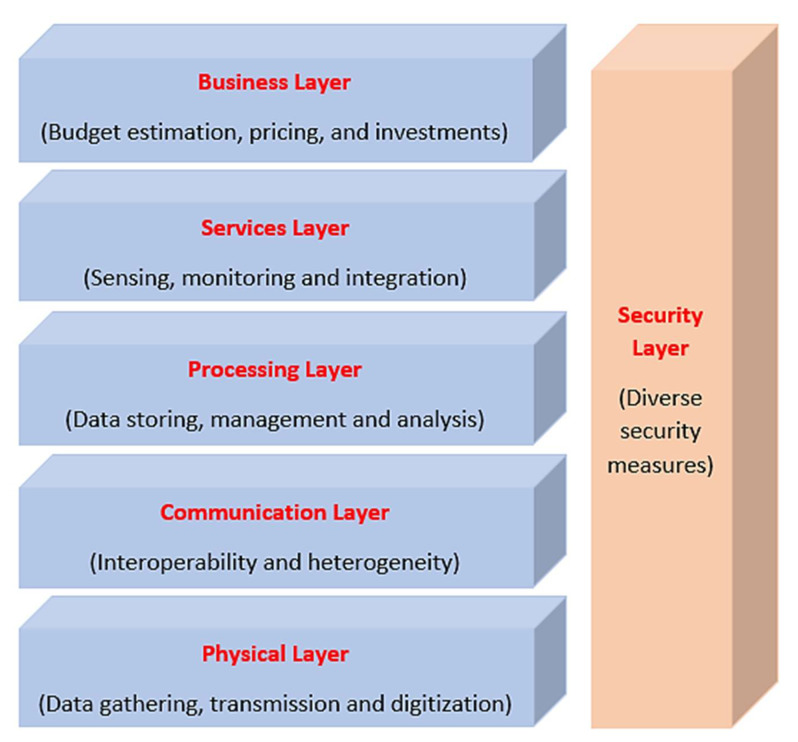
The proposed six-layers architecture of IoV.

Different layered architectures for IoV frameworks have been proposed in the literature, which differ in the number and/or names of layers proposed. These architectures compete to optimize the number of layers and to enhance the distinguishability between the different layers [57,58,60,61,62,63]. The most common IoV architecture can be seen in Figure 2 which defines six layers, namely, physical, communication, processing, services, business, and security. The main responsibility of the physical layer is to gather information about vehicles and their surrounding environment such as vehicle’s speed, position, travelling direction, on-road vehicular density, weather conditions, and others through the sensing devices, actuators, GPS modules, and access points installed on the vehicles. RSUs and other personal devices may also be used. The collected data are then transferred in a secure way to the processing layer through the communication layer, which employs diverse wireless communication standards and network modules to guarantee interoperability between the different heterogeneous network entities such as WAVE, WiFi, RFID, Bluetooth, 4G/LTE, UW, and satellites. The processing layer represents the storage, processing, and transformation of the data received from the lower layers into useful information to be used for decision making. This includes the adoption of various big data analytics tools and cloud computing platforms. The services layer then takes the information processed and the decisions made by the processing layer and employs them to provide intelligent IoV services and applications to the end users which can contribute to road safety and traffic efficiency. The business layer’s responsibilities can include decisions related to economics investment, budget estimation and regulation, pricing, and operations management. Finally, the security layer concerns about secure and reliable data collection and communication among the different nodes to prevent against diverse number of security attacks and threats that can be encountered in IoV environments. Since security is the main theme of this paper, we will discuss more on this in the coming sections.

### 2.2. Blockchain

A blockchain, also called a Distributed Ledger Technology (DLT), can be defined as a group of connected blocks used to store transactions’ records or events and is maintained by all the participating users distributed over the network. Blockchain technology removes the reliance on a central authority, since it allows all users to generate and validate transactions directly in a peer-to-peer network which helps significantly in reducing the financial and time related costs associated with the intermediate party. Building the blocks of a blockchain relies on two key technologies, namely, cryptography [64,65,66] and consensus mechanisms [67,68,69].

Cryptography is adopted in blockchain to ensure the security and privacy of the data and the anonymity of the participants thereby using cryptographic hash functions and digital signatures [70]. The adoption of cryptographic hash function is quite popular in blockchains, where each block is linked to the previous block (its parent block) by keeping the hash value of the previous block in its own header, except the genesis block, i.e., the first block in the chain. The first block has no parent block and thus the hash value of the previous block is set to zero. This hash-based linking structure makes the blockchain immutable due to the uniqueness of the hash values used. The digital signature, on the other hand, is a type of asymmetric cryptography where each user owns a pair of public and private keys. The typical digital signature algorithm used in most blockchain applications is the Elliptic Curve Digital Signature Algorithm (ECDSA).

Consensus mechanisms are used in the blockchain to establish trust in an untrustworthy environment and to verify the correctness and integrity of the transactions data being added to the public ledger. Blockchain consensus can be defined as “the agreement of a common value among a group of nodes in blockchain systems” [71]. Several consensus mechanisms have been proposed in different blockchain scenarios which differ in their fault tolerance, scalability, power consumption, and application-dependent scenarios. However, they all agree to provide consistency and transparency to the data blocks. Two broad categories of blockchain consensus protocols are suggested in [67], namely, probabilistic-finality consensus and absolute-finality consensus protocols. The former can only guarantee an eventual consistency whereas the latter ensures a strong consistency. Some of the most common consensus protocols are Proof of Stack (PoS), Proof of Work (PoW), and Practical Byzantine Fault Tolerance (PBFT). However, other less common consensus mechanisms also exist in blockchain applications such as Leased Proof of Stack [72], Proof of Elapsed Time [73], Proof of Activity [74], Proof of Importance [75], Proof of Capacity [76], Proof of Burn [77], and Proof of Vote [78].

Three types of blockchains are commonly defined and agreed upon by most of the literature, namely, public blockchain, private blockchain, and consortium blockchain. The different types are distinguished from each other by their consensus making, read permission, immutability, and degree of centralization [68]. Public blockchain is a non-restrictive, permissionless distributed ledger in which everyone can access and validate the transactions and participate in running the consensus mechanism. Public blockchains are completely decentralized and are suitable for fully opened systems where untrusted entities may join the network. Typical examples of public blockchains include Bitcoin, Ethereum, and Litecoin [69,79,80,81]. On the other hand, Private blockchain is a restrictive, permissioned blockchain in which only a sub-group of predefined nodes can maintain and validate the ledger. A private blockchain is fully controlled by a single organization, thus it can be regarded as a centralized network. Private blockchains are suitable for closed systems where all nodes fully trust each other. Consortium blockchain is a partially decentralized ledger managed by several organizations in which only a small group of nodes is pre-selected to perform the consensus. It is suitable for semi-closed systems consisting of few enterprises and thus is normally found in the banking sector and other governmental organizations. Consortium blockchains are regarded as a combination of both public and private blockchains. Typical examples are Stellar [71], R3CEV [79], Hyperledger [82], and Ripple [83].

However, other blockchain classifications have been suggested in the relevant literature. For instance, the authors in [69] have divided blockchains into four types: public, private, consortium, as well as hybrid. Similarly, two blockchain categories, permissioned and permissionless blockchains, are proposed in [84].

### 2.3. Motivations to Use Blockchain in IoV

IoV is a large-scale and heterogenous network that combines a large number of connected vehicles, roadside infrastructure, mobile personal devices, central and distributed storage, and computation servers in case of incorporating cloud and edge computing platforms. This along with the open-channel wireless communication model and the public Internet connectivity that dominate most of the communication makes the IoV network vulnerable to a variety of security attacks that could threaten the applications of IoV such as navigation, accident detection and notification, dynamic alternative routing, route optimization, and congestion management which consequently constitutes a threat and danger on drivers and passengers on the road. Furthermore, since IoV scenarios include high mobility and exchange of huge amount of data as well as requiring real-time services and decision making, more efficient, powerful, and reliable technologies must be adopted in IoV frameworks in place of the conventional techniques. 

On the other hand, blockchain technology has emerged recently as a decentralized storage mechanism in various industry applications due to its strong capabilities not only in distributed storage aspect, but also in terms of security, privacy, performance, automation, and reduced computational cost. Recently, blockchain has also been brought to the IoV paradigm to serve different purposes such as data protection and management, resource trading, resource sharing, ride sharing, traffic control and management, and forensic applications. 

The various features a blockchain can provide have motivated researchers and the industry to incorporate blockchain technology into the IoV. These properties include the following [85,86]:

1. Decentralization: Unlike the centralized-storage platforms where both data storage and management are handled by a trusted centralized node, blockchain technology exhibits a decentralized nature in which data records are kept and managed by all participating entities. This reduces the resource bottlenecks issue and maintenance cost associated with the centralized sever arrangements and avoids the single point of failure issue which all can be beneficial for IoV environments.
2. Immutability: Since the creation and validation of new blocks of transactions should be agreed upon by all or most of the peers via the different consensus mechanisms before being added to the blockchain, the blockchain is almost impossible to be tampered with or modified.
3. Security and privacy: The cryptographic nature of blockchain where both cryptographic hash functions and digital signatures are adopted can ensure the security of transactions data and the privacy of the participating users in IoV.
4. Transparency: Since all participants keep a replica of the public ledger, they can access all the timestamped blockchain transactions. This enables the peers to manage, look up and verify transactions at any time in a transparent manner without an intermediary. This self-auditability and transparency not only promote the relief of the peers by managing their own transactions, but also mitigates the time and financial costs associated with the intermediate party.
5. Automation: Blockchain technology supports the adoption of smart contracts which are software scripts that can be executed automatically by a triggering event or upon meeting some pre-defined set of rules. This automation property of blockchain can enhance the efficiency of many IoV applications and help provide various services autonomously without a need for a trusted entity.
6. Traceability: Each transaction record is kept in the blockchain with a timestamp indicating its time of occurrence and joining the public ledger. This timestamped recording nature helps identifying the events in a chronological order which enhances the traceability and can support the non-repudiation requirement in IoV.

## 3. Security Issues in IoV and VANETs

IoV and VANETs have various features that are advantageous to vehicles’ drivers and passengers, pedestrians as well as the whole industrial business. However, like any new-emerging technology, IoV and VANETs come with several risks and security threats. The continuous mobility of vehicles, the existence of a third party acting as an authority to certify the nodes, and the wireless mode of communication among the different nodes make these vehicular networks vulnerable to wide range of security threats and attacks. Identifying the different security requirements and exploring the possible attacks that threaten these vehicular frameworks is the first step towards resisting them. Accordingly, in this section we first present the different security requirements and challenges for vehicular networks in Section 3.1 followed by a wide range of security attacks in Section 3.2.

### 3.1. Security Requirements and Challenges in Vehicular Networks

The IoV networks are an amalgamation of diverse technologies with different standards and regulations (such as Internet connections, different wireless technologies, sensors, cloud services); which make IoV vulnerable to various types of security attacks. Depending upon the attacker objective(s), the attack launched might be passive or active, generated internally or from an external source. However, regardless of the attack’s source or activity type, these security threats are commonly classified into different categories based on the security aspect(s) of the network being compromised. For example, an attack could affect the authenticity of the users, the integrity of IoV data, or the availability of the provided services. 

**Figure 3:**
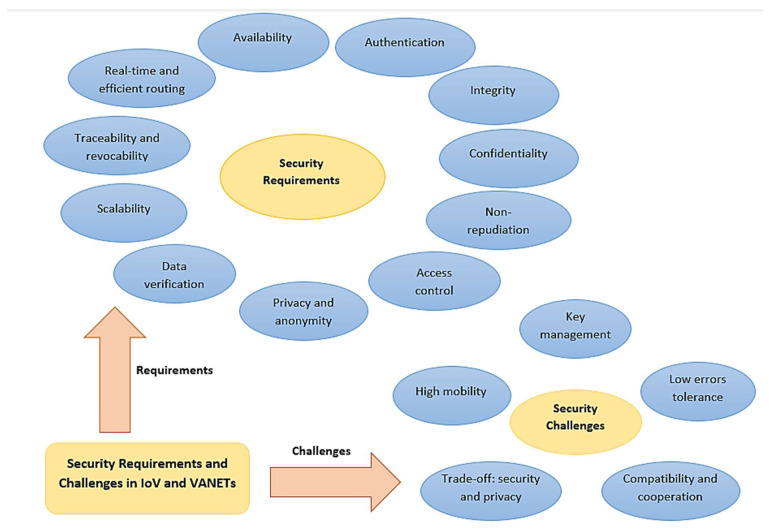
The security requirements and challenges in IoV and VANETs platforms.

Guided by the various security threats an IoV system can suffer from, different security aspects have been defined in the literature. These security aspects can be classified into: (1) Security requirements that an efficient IoV system should maintain and (2) Security challenges that face any security subsystem of an IoV environment. Figure 3 illustrates these security requirements and challenges. Following are the different security requirements of an IoV system [87,88,89,90,91]:

1. Authentication: It means ensuring that the received data is generated by a legitimate sender, or in other words, making sure that the entity that sent the data must be the true actual entity it claims to be. It guarantees that the entities involved in the communication are authentic, not masqueraded by some attacker who forwards the messages on their behalf. Sybil attack, masquerading attack, impersonation attack, spoofing attack, replay attack, and wormhole attacks are examples of attacks that target the authenticity of IoV users.
2. Availability: It is a basic requirement in IoV environment especially for the real-time critical applications where even a minor delay cannot be tolerated and made the information useless. Therefore, the IoV system should be available all the time to provide real-time information and services to all legitimate users and be able to tolerate partial system faults and failure issues through backups and replications. Moreover, a mature IoV system must have the ability to function under intense network load with the increasing number of participants. The common attacks that target the availability service are the denial-of-service and distributed denial-of-service attacks.
3. Confidentiality: Some IoV applications include sensitive information that are accessed only by certain legitimate users. Therefore, confidentiality of this information must be insured through encryption to prevent it from being revealed and interpreted by any illegal entity even upon eavesdropping.
4. Data integrity: It means there is no distortion—whether intentionally or accidentally—in the received data. In other words, the sent data and the received data are identical. Typical attacks on integrity can be data manipulation attack and malware attack.
5. Non-repudiation: It guarantees that any involved user in the IoV environment cannot deny any of its past activities, i.e., sending or receiving any piece of information. This ensures that an attacker can be identified, and all its communicated messages can be retrieved if needed for subsequent actions.
6. Access control: Each participating entity in the IoV network is assigned different rights and privileges to access the network resources. This security requirement guarantees that each node performs its functions based on the services it is entitled to.
7. Privacy and anonymity: The users’ real identities may need to be made hidden using anonymous identities or pseudonyms to protect their privacy. Additionally, some location information such as the driving traces and tracks followed by the vehicles are sometimes preferred to be anonymous in order to prevent unauthorized location tracking.
8. Data verification: Since malicious entities can modify the information sent by the sender, a regular data verification process is usually performed to identify the manipulated or tampered messages and thus reject or drop them (if found) to prevent misleading the receiving entities into taking improper decisions.
9. Real-time guarantee and efficient routing: The majority of IoV and VANETs applications are real-time, such as accidents detection and warnings dissemination, which must be carried out within certain time constraints, otherwise the safety of drivers and passengers could be threatened, and the delayed information will become worthless. To be able to meet these time constraints, efficient secure routing protocols should be adopted to guarantee delivering the packets in their entirety and on time.
10. Traceability and revocability: Despite the need for preserving the privacy of IoV users in general by hiding their real identities, the legal authorities should have the ability to retrieve the vehicles’ real identities in case of misbehaving to revoke them as well as in case of disputes.
11. Scalability: With the increasing number of vehicles on the roads nowadays, more vehicles and entities are joining the network. Thus, a good vehicular network should be able to scale-up accordingly. However, this nodes extension may expose the network to higher security issues if not controlled and monitored properly. Therefore, monitored scalability is another security requirement of the IoV system.

The following are different security challenges that might be faced by any IoV paradigm [89,92,93,94,95,96,97]:

1. High mobility: Data packets must be preserved and kept unmodified during the entire uncertain routing process from the sender to the receiver and should also be delivered on time to satisfy the real-time security requirement. However, the high mobility of nodes in IoV and VANETs networks, and the continuously varying network topology have led to the transient nature of V2V and V2I interactions, which makes the real-time guarantee and non-repudiation security requirements much more difficult.
2. Low errors tolerance: Any minor delay in receiving information or delivering packets in IoV may result in unacceptable situations or even road disasters, thus the time constraints are of high importance in such environments. However, the limited bandwidth and the unstable network quality in IoV caused by the huge number of vehicles being served, their mobility, and the unpredictable changes in wireless networks hinder the real-time security requirement. Therefore, some preventive security measures must be applied so that the drivers can be proactive in case of any emergency situations.
3. Key management: Several authorities and stakeholders are considered important participants in IoV such as governmental institutions and vehicle manufacturers. Hence, it has always been a security challenge to delegate a certain authority among these stakeholders to serve as a fully Trusted Authority (TA) that is responsible for key distribution, management, and revocation, since choosing the wrong authority without considering its hidden intents and benefits could severely affect the security of the IoV system. Moreover, due to the scale of IoV and VANETs networks, the Certificates Revocation Lists (CRLs) that are responsible for the revocation of misbehaving and malicious vehicles become too long and thus not feasible which raises the overhead of the certificate revocation process. Thus, maintaining a balance between efficient key distribution/revocation process and low overhead is another challenge on IoV security.
4. Tradeoff between security and privacy: In general, the more secure the system is, the less privacy it can provide for users. Many drivers and passengers may not be willing to sacrifice their privacy by sharing their private sensitive data such as their location and destination while caring for security at the same time. Thus, maintaining a good balance between high security and reasonable user privacy is another major security challenge in IoV.
5. Compatibility and cooperation: Due to the divergent interests and targets of the various IoV participating parties such as vehicle manufacturers, consumers, and governmental organizations, it is a big challenge to align their interests properly.

### 3.2. Security Threats and Attacks in Vehicular Networks

As stated previously, different types of security attacks can be launched against IoV or VANETs, whether passive or active, insider or outsider. However, the common factor of all of them is that they constitute a serious threat to the security of the users, the integrity of the flown data, and consequently the safety of the vehicles’ drivers and passengers. Various security attacks and threats in vehicular networks (i.e., IoV and VANETs) have been defined in the literature [89,95,98,99]. Following is some of the most common ones:

- Channel Interference Attack: This is also known as jamming attack, which is the act of interfering with the wireless communication links to hinder and disrupt the quality of communication between connected IoV devices.
- Denial of Service (DoS) Attack: In this attack the attacker floods the IoV network with fake unnecessary requests so that the service provider is no longer able to handle the legitimate requests issued by the genuine users.
- Distributed Denial of Service (DDoS) Attack: It is an advanced type of DoS attack, in which the fake requests are launched from several distributed nodes forming botnets.
- Man-in-the-Middle Attack: In this attack, an attacker places itself in the middle of two communicating legitimate entities to receive the data from the sender and then forward it to the receiver. When the message passes through the attacker, he/she could modify it before forwarding it to the receiver or just reveal its content.
- Message Tampering Attack: This attack aims to corrupt the messages and spread false information in the network by manipulating and fabricating the contents of the communicated messages. This can badly affect the road safety and the services provided by IoV thereby harming passengers and drivers.
- Eavesdropping Attack: It is also referred to as traffic analysis attack, in which the attacker monitors the network to track vehicle routes for some malicious purposes. The attacker eavesdrops on the communication channels to examine the messages flowing in the network.
- Malware Attack: In this attack, the attacker injects malicious software or viruses in the system files to contaminate the network, which can be leveraged to disrupt the network services and/or to manipulate users’ data.
- Message Holding Attack: Here, the attacker drops some sensitive messages that hold important data about road conditions that could greatly affect the decision of the drivers on road and thus the road safety is compromised. It also enables the drivers to keep these messages for future use (replaying) in the network.

The above-defined attacks are examples of common security threats that can affect the confidentiality and integrity of IoV data, as well as the availability of the entire IoV system. Table 2 depicts a mapping between the various presented security attacks and the security requirements affected by each of them. However, since our target in this survey paper is to review and discuss the authentication-related issues in IoV, we dedicate more focus to the attacks and threats that target the authentication domain specifically. Therefore, Table 3 illustrates the authentication attacks with their targeted OSI layer along with some proposed solutions, and following are their definitions:

- Sybil Attack: An adversary creates multiple fictitious vehicles on the road from a single node/vehicle by allocating it different identities to appear as it represents separate entities. In other words, an attacker creates (and then controls) more than one identity on a single physical node. This implies that other entities in the network will not be able to judge if the data originate from a single vehicle or multiple vehicles. This attack can be used to affect the route selection of a target vehicle by creating a fake traffic jam on the road which forces the targeted victim vehicle to choose an alternative path.
- GPS Deception/Spoofing Attack: It refers to the interception and manipulation of the GPS signals while being sent between two legitimate IoV nodes, to mislead the receiving entity by providing wrong location information about the sending vehicles. This can directly harm the drivers and the passengers by affecting their path decisions. Additionally, when a GPS receiver of a vehicle is attacked, it could provide the vehicle with false information about its location, speed, and other GPS information, which can greatly affect some IoV applications that depend on this GPS information such as navigation devices.
- Masquerading/Impersonation Attack: A node pretends to be another node by stealing its identity. Here, both the legitimate vehicle and the adversary vehicle can use the same identity simultaneously which can create a chaos in the network since other vehicles may receive contradictory information from the same identity. The malicious node can also enjoy the access privileges of the spoofed identity.
- Worm Hole Attack: Also known as tunneling attack, refers to the attack where at least two malicious vehicles create a private tunnel known as a worm hole which is used to forward the intercepted data between the two of them [89]. In this attack, the true distance information is faked where other entities are forced to route through the created tunnel thus controlling all the traffic flowing in the network.
- Replay Attack: In this attack, an adversary keeps iterating the old messages in the network to deceive the other nodes by dropping the messages with high priority from the queue. This can greatly affect the system’s efficiency and increase the cost of the bandwidth.

## 4. Blockchain-Based Authentication Schemes in Vehicular Networks

The focus of this survey as mentioned previously is on blockchain-based authentication mechanisms in vehicular networks. We surveyed a wide range of the existing schemes in the literature and categorized them based on the type of the blockchain used for authentication into three categories: (a) private, (b) public, and (c) consortium blockchain-based authentication. These categories are discussed below.

### 4.1. Private Blockchain-Based Authentication Protocols

A private blockchain is a limited access blockchain in which only a particular group of trusted entities (which is decided by a network administrator) is granted access permissions to the blockchain transactions for performing specific tasks.

The nature of private blockchains enables establishing a high level of trust during authentication since only a small group of trusted nodes are allowed to access the vehicles’ authentication parameters stored in the blockchain. Additionally, being controlled by a single organization allows easier, more efficient management and supervision over the authentication data kept at the ledger. Consequently, private blockchains are implemented by many researchers to serve authentication purpose in IoV platforms.

The authors in [111] incorporated a private blockchain technology with intelligent contract to address the issue of new nodes joining the IoV network. The intelligent contract is designed initially for the verified cloud servers, roadside units and vehicle manufacturers which form a contract node group that use Rayleigh consensus mechanism to authorize or reject the new joining requests. When a new node sends a registration application to join the contract group, each node in the contract node group evaluates the received application and grants its digital signature if it agrees to trust the node, otherwise no digital signature is granted. Then, if 51% of the contract nodes grant their signatures, the node is accepted to the contract node group and a new block is added to the chain. Otherwise, the node’s information is added to the list of suspicious nodes and broadcasted to the rest of the network to block its future joining attempts. This results in suppressing the joining of malicious nodes from the root. 

The authors also addressed the authentication of registered vehicles by adopting a Public Key Infrastructure (PKI) technology based on cryptographic accumulator to enhance the authentication efficiency. The authentication process is performed in two phases; the first phase is to verify the vehicle’s identity to the roadside unit, and the subsequent phase is the mutual verification between the RSU and the cloud server. In the first phase, the vehicle sends its ID and public key to the RSU which verifies the vehicle identity and adds its own public key to the vehicle information and sends them to the corresponding cloud service provider authority (CA) after encryption with the public key of this CA. Upon receiving the message from the RSU, the CA starts the second phase by first decrypting the message with its own private key, and then searches the vehicle ID and the public key of the RSU in the blockchain to check whether the provided information is correct. If successfully found, a new session key is generated by the CA and sent to the RSU along with the corresponding vehicle ID after encryption with the RSU public key. The RSU decrypts the packet with its private key, stores the session key for securing further communication with the CA, and sends the issued digital certificate to the vehicle encrypted with its public key, upon which the authentication process is culminated.

The proposed solution is evaluated using Veins open-source framework through a small-scale network of 13 nodes in terms of the time overhead and communication cost needed for the encryption process related to the blockchain technology and for the whole authentication process. Their scheme proves to provide high authentication efficiency in preventing malicious attacks with low time and communication costs. However, large packet loss is encountered during the registration of the vehicles and the key distribution process.

The authors in [112] discussed the issue of computing and communication bottlenecks faced by the centralized authentication protocols and single Trusted Authority (TA) schemes that could fail to authenticate the large number of simultaneous vehicle requests within a limited time during high mobility. Thus, they suggested a blockchain-based RSU-assisted authentication and key agreement protocol for a multi-TA network model by offloading part of the authentication process to the RSUs to achieve more decentralization. The aim of offloading is to reduce the resource bottlenecks of the TAs, which results in improving the authentication efficiency. The blockchain technology is used to address the cross-TA authentication issue which is caused by the high mobility of the vehicles in a multi-TA environment. Instead of restarting the whole authentication process when a vehicle exits the coverage area of one TA to the coverage area of another TA, the blockchain adoption allows the new TA to continue the authentication process that was started by the old TA since they manage the same ledger that keeps all vehicle-related information.

The network model consists of four types of nodes: vehicle nodes (VNs) and RSUs which are both considered as untrusted nodes in their threat model, TAs which are assumed to be semi-trusted nodes, and a data center (DC) which is assumed as a fully trusted network entity that stores all vehicle-related information. A smart contract is used to automate part of the authentication process and the delegated proof-of-stack [113] is adopted as a consensus mechanism for more efficient resource utilization and power consumption. The whole scheme is composed of three phases, namely the initialization phase, the registration phase, and the authentication phase. In the initialization phase, the system administrator generates a master key and sends it to all RSUs and TAs. Each VN must be registered with the nearest TA during the registration phase in which a unique ID is granted to it and kept as a record with a unique pointer P in the ledger as well as in the vehicle’s memory. The pointer P is then broadcasted to all TAs which jointly pack it into a new block and link it to the previous block in the chain.

The authentication phase has five steps. In the first step, the VN enters the RSU communication range and issues an authentication request to the RSU. Then, the RSU forwards a part of the request message to the TA in the second step asking for the VN-related authentication parameters. In the third step, the TA executes the smart contract in the blockchain. It checks first whether P exists in the blockchain, then retrieves the VN-related authentication parameters from the DC according to P, and then sends these parameters to the RSU. Once the RSU successfully authenticates the TA and the VN, it sends the updated parameters to the VN and the TA in the fourth step. In the final step, after the TA successfully authenticates the RSU, it sends the updated parameters to the DC via the secure channel. Once the DC has updated the parameters, the TA sends acknowledgment message to the RSU. After the VN successfully authenticates the TA, it updates the authentication parameters in its memory. After the VN and the TA negotiate a session key, it remains valid as long as the VN lies within the communication range of the RSU. Once the VN leaves the communication range of the RSU, the session key will be revoked.

A detailed security analysis is performed on the proposed scheme using ProVerif tool [114] to check its robustness against different security attacks. The results prove that the scheme can resist eavesdropping attack, replay attack, VN impersonation and VN capture attacks, TA and RSU spoofing attacks, and jamming attack. It also guarantees backward/forward secrecy and VN anonymity.

The authors in [115] proposed a protocol termed as “BlockAPP” which serves for both authentication and privacy preservation of vehicles identities. The system architecture contains a registration server and multiple service providers. The registration server is responsible for validating and managing vehicles identities whereas the service providers perform the authentication process. The registration server can only write to the blockchain whereas the service providers have both read and write permissions on the blockchain. Further, two types of blocks are defined within their blockchain, i.e., one is created by the registration server to keep a log of the registered vehicles and the other is added by the service providers to keep track of the access history.

The proposed scheme has three phases: the registration phase where the vehicles interact with the registration server, the authentication phase and the authorization phase which are handled by the different service providers. The registration phase starts when the vehicle sends a registration request message containing its original id (i.e., driver’s license or vehicle registration number) to the registration center and then a key exchange process takes place using the Elliptic Curve Diffie Hellman (ECDH) key exchange protocol [116] to exchange their public keys. After which the registration server generates a pseudo id by encrypting the vehicle’s original id with its session public key and sends it to the vehicle. Upon receiving an acknowledgement from the vehicle, the registration server adds a transaction with the vehicle-related information to the blockchain after validation. A vehicle then sends a message containing its pseudo id and the session parameters obtained from the previous phase to a service provider which authenticates the vehicle’s identity by comparing the received data against the vehicle’s information kept in the blockchain. If matched, the vehicle is successfully authenticated, and an access log transaction is added to the blockchain by the service provider. The vehicles can then apply for various services during the authorization phase by sending a service request message with the digital signature of the service. The use of pseudo ids for authentication instead of original ids while restricting their validity to only one session, not only protects the privacy of vehicles but also prevents the system from identity spoofing attacks.

The authors in [117] suggested a secure mutual authentication scheme with reduced dependency on certification authority (CA) by introducing a private blockchain framework. In addition to vehicles, the physical entities involved in their model are the CA and the revocation authority (RA) which both have complete control over the blockchain. The RSUs, on the other hand, have only read permission over the blockchain.

The scheme is composed of three phases, namely, system initialization, registration of the vehicles, and mutual identity authentication and revocation. In the first phase, the CA initializes the system parameters including the elliptic curve parameters and hashing functions. Moreover, the public key pairs are generated and transferred to the blockchain network entities, i.e., CA, RA, and RSUs. When a vehicle first registers with the CA, it submits its original vehicle id obtained from the motor vehicle’s division. The CA verifies the vehicle’s original id and assigns for it a Pseudo Id (PID) along with the Elliptic Curve Cryptography (ECC) public–private key pair. The PID is then signed digitally by the CA using the Elliptic Curve Digital Signature Algorithm (ECDSA) [118], forming a new transaction which is added to the blockchain under the proof-of-authority consensus mechanism among the multiple CAs. When the vehicle’s registration information, i.e., PID and the digital certificate, is added successfully to the blockchain, a pointer referring to its storage location in the blockchain along with a unique transaction id are sent back to the CA. At this point, the CA transfers the pointer, the PID, the certificate along with the ECC key pair to the vehicle’s OBU. The CA also stores a record mapping the real identity of the vehicle to the Pseudo identity in the hash table in its local database which helps facilitate the lookup in the case of traceability and revocation of malicious vehicles.

When the vehicle becomes active on the road, it initiates an authentication request containing its PID, hash pointer, and transaction id to the nearest RSU. The RSU then uses the received PID as an index to query the blockchain for the vehicle’s respective transaction, if verified, the RSU sends a challenge message to the vehicle encrypted with its public key and waits for the reply. If the vehicle successfully decrypts the challenge message and sends the correct response, it is authenticated successfully by the RSU. Extensive simulation using Vein’s framework and OMNeT++ network simulator proves the efficiency of this scheme in terms of authentication delay, transaction throughput, and packet-delivery ratio.

The authors in [119] proposed a distributed message authentication scheme using a private blockchain, where vehicles can authenticate the messages broadcasted in the network in a distributed manner. The system model is composed of a single Root Trusted Authority (RTA), multiple Local Trusted Authorities (LTAs), RSUs, and vehicles. The RTA is a fully trusted authority that is responsible for managing the entire system and performing the registration process whereas the individual LTAs are responsible for authenticating the vehicles in their local areas. The authors define two types of nodes based on the task they perform on the blockchain, namely, block generation nodes and block verification nodes. The generation of blocks is performed through the proof-of-work consensus mechanism and is assigned to the infrastructure nodes, i.e., LTAs due to their high computing capability, while the verification of the blocks is the responsibility of the vehicles. Since the vehicles are highly mobile and have a relatively low computing power, the consensus during block verification must be completed quickly, and thus the use of the Practical Byzantine Fault Tolerance (PBFT) consensus mechanism.

The proposed message authentication scheme includes six phases: initiation, registration, message signing, message verification, block generation, and block confirmation. In the initiation phase, the RTA generates its public–private key pair, the genesis block of the blockchain and the list of LTAs. During the registration phase, a vehicle’s owner sends the vehicle’s information and its biometric information to the RTA which in turn generates a pair of asymmetric keys and transfers it along with the system key to the vehicle’s local memory. The registration of LTAs also takes place in this phase by the RTA in the same approach as the vehicles’ registration. When a vehicle needs to broadcast a message into the network, it computes the message’s hash value h(m) and concatenates it with the hash values of the previous blocks in the blockchain and encrypts them together with its private key. This arrangement is then attached as a header to the actual message content along with a timestamp and encrypted with the system key before being broadcasted to other vehicles. This process of message signing guarantees the legitimacy of the sending vehicle and thus the authenticity of the broadcasted information since the unauthorized vehicles cannot have the system key used for message signing as it is distributed only to the registered vehicles during the registration phase.

As the V2V messages are broadcasted among the vehicles, the corresponding LTA keeps collecting those messages until it forms a block after a certain number of messages. The block is then verified through the PBFT consensus by the vehicles and their LTA. Once the block is verified, the LTA transfers it to the other LTAs for block confirmation process. If confirmed by all LTAs, they send the confirmed block to the RTA which then concatenates the block officially into the blockchain. The simulation results reveal that the proposed scheme can prevent impersonation attacks and single point of failure issue while providing a highly efficient blockchain-based authentication in terms of the total consensus delay and throughput.

The above-mentioned private blockchain-based authentication schemes can be found in Table 4 for easy reference.

### 4.2. Public Blockchain-Based Authentication Protocols

A public blockchain is an open access blockchain in which everyone can access, send, receive, and verify the different transactions of the blocks. Since it is a fully opened blockchain, even the vehicles can participate in the authentication process by looking up the ledger for the targeted authentication parameters. This provides a better utilization of the available computing resources in the IoV environment compared to restricting the authentication process to a few trusted nodes, thus a more decentralized, time efficient authentication process is achieved. These characteristics of a public blockchain have attracted many researchers to adopt it for IoV authentication.

The authors in [120] contributed to address the authentication delay issues and time complexity in IoV network. The proposed work provides real-time authentication and adversary detection through the adoption of a public blockchain. The authors used the wireless channel characteristics, such as the received power and the Link Fingerprint (LF) along with the hash technology of blockchain to detect any intrusion in V2V communication in real-time. The main concept is that the wireless link between any two communicating vehicles has a unique fingerprint which is generated from the channel’s power characteristics. As a result, the variation in the received power of the communicating vehicles must highly correlate, otherwise the communication path is intercepted, and an adversary is detected. Each vehicle uses the LF, a pseudo-random freshness parameter N, (that changes every time to prevent the system from replay attacks), and the hash of the previous block used to generate the hash value. The hash is then stored in its local memory and sent to the cloud to be stored in the publicly accessible ledger. A sending vehicle encapsulates the packet with a header containing its hash value before being sent to the receiving vehicle. When the receiving vehicle gets the packet, it removes the packet header and looks up the hash value in the ledger to check the authenticity of the sender vehicle, if it exists, it accepts the package data; otherwise, an adversary is detected, and the packet is discarded. 

MICAz mote—a hardware wireless sensor module—is installed on two vehicles to serve as a wireless communication interface in the V2V arrangement. Measurements are recorded indoor and outdoor in real-time and reported with the aid of MATLAB R2020a. The Pearson Correlation Coefficient [121] is computed to detect an adversary in the network when its value is less than or equal to 0.9. The scheme is also evaluated in terms of time complexity which is found to be as low as O(1) due to the simple and lightweight hash function used in their blockchain.

The authors in [122] proposed a scalable blockchain-based protocol that deploys a dynamic proof-of-work consensus mechanism and Physical Unclonable Functions (PUFs) for authentication and trust establishment. Their approach depends on the assumption that the smart vehicles are equipped with PUF components that generate hardware fingerprints which serve as unique identities replacing passwords and secret keys. The authentication process passes through two phases, namely the setup phase and data transfer phase. The vehicle’s registration process is performed during the setup phase, where each vehicle is given a pair of public and private keys and allocated an account address in the blockchain. A smart contract named “enforcer” is utilized to initiate the communication between the vehicles and the blockchain whereas the RSUs serve as the blockchain miners and the certificate authority as well. When a vehicle generates data traffic, the enforcer first checks its existence in the list of registered vehicles stored in the RSUs. If it is registered, a PUF challenge is then sent to the vehicle. If the vehicle succeeds in this challenge, the authentication is completed successfully, and a communication link is established between the vehicle and the local blockchain. A digital certificate is then issued by the RSU to the vehicle to serve anonymity and privacy preserving purposes for future authentication process.

After registration and successful authentication, a vehicle can communicate with other entities in IoV environment and exchange data in the data transfer phase. The deployed dPoW consensus mechanism allows the protocol to scale based on the incoming traffic generated by the vehicles and their use of PUFs makes the vehicles immune to physical and impersonation attacks. A detailed analysis is conducted through software implementation and NS3 simulation tool to evaluate their scheme in terms of the four-way tradeoffs of distributed systems which are scalability, decentralization, security, and latency. Two types of delay were measured: (1) the authentication delay at the RSU, which is time needed to authenticate a vehicle by the RSU and (2) the time to finality, which is the time needed to form a block, mine it, and reach a consensus on the mined block. The results show that their authentication scheme efficiently satisfies all the above-mentioned four properties without sacrificing any of them.

The authors in [123] designed a novel blockchain-assisted authentication scheme for Artificial Intelligence (AI)-envisioned IoV-enabled smart cities called “BBAS-IoV” by which authentication is performed both individually and in batches. The network model is composed of several smart cities; each is managed by a separate Trusted Authority (TA) and divided into multiple clusters. Each TA is responsible for initializing the system parameters during the initial setup phase, registering RSUs and vehicles within its service area during the registration phase and distributing certificates and private/public key pairs later to them. The model contains a fog server connected to each group of geographically related RSUs and a group of cloud servers forming the publicly accessible blockchain center.

Beside the setup and registration phases, the protocol has other six phases, namely, message signing, authentication, group key management, blockchain formation, AI-based secure big data analytics using blockchain and dynamic nodes addition. During the authentication phase two types of authentications exist, i.e., V2V and batch authentication. The V2V authentication enables each vehicle to authenticate its neighbors in its cluster in the smart city. The batch authentication is then used by the RSU to authenticate all its clusters’ vehicles simultaneously which saves time and reduces the computation overhead of the whole scheme. After that, a group key is agreed upon and granted to each cluster to be used for securing communication among intra- and inter-cluster vehicles and their managing RSU. The blockchain formation phase is handled by fog and cloud servers in two steps; first, each fog server receives the list of transactions and their compact signatures from the associated RSUs and verifies them, if the signatures are valid, it transmits the partial blocks to a cloud server in the blockchain center. Then, the cloud servers convert the received partial blocks into complete blocks which are then mined and voted for through the PBFT consensus mechanism to decide on their eligibility to be added to the blockchain. The signature verification and block verification applied in this scheme ensures that data poisoning attacks which inject malicious transactions in the blockchain are avoided in the proposed BBAS-IoV protocol. This results in fully trusted blockchain transactions that can be a great asset for deriving highly accurate ML models and thus correct predictions and decision making can be achieved.

Detailed formal security analysis using the Automated Validation of Internet Security Protocols and Applications (AVISPA) tool as well as informal security analysis is provided to evaluate the security features of the proposed BBAS-IoV scheme. The results obtained show that various security attacks namely, replay attacks, man-in-the-middle attack, vehicle and RSU impersonation attacks, privileged-insider attack, and ephemeral secret leakage attack are prevented. Extensive performance evaluation through simulation with the aid of Multi-precision Integer and Rational Arithmetic Cryptographic Library (MIRACL) reflects high efficiency of the scheme in terms of computation, communication, and storage overheads.

The authors in [124] designed a blockchain-based authentication scheme for vehicular accident detection and notification in IoV-enabled intelligent transportation systems, called BCAS-VADN. The scheme deploys a cloud/edge computing framework that consists of cloud servers, edge servers, RSUs, and vehicles. The system architecture is divided into multiple clusters with a Cluster Head (CH) for each, which acts as an intermediate entity to arrange the communication between the cluster members and the RSUs. Each RSU is associated with an edge server and all edge servers are linked to cloud servers in the blockchain center through a public channel. The proposed scheme consists of five phases, namely, system initialization, enrollment, authentication, blockchain verification and addition, and dynamic node addition phases.

The proposed scheme assumes the existence of a trusted registration authority that is completely responsible for initializing the system parameters including elliptic curve, one-way hash function and its own private–public key pair as well as enrolling all the entities in the network, i.e., cloud servers, edge servers, RSUs, and vehicles. The authentication phase then takes place in two steps: V2CH authentication in which a vehicle and its associated CH mutually authenticate, then CH2RSU authentication where the CH and its associated RSU authenticate each other. Upon a successful authentication process, each vehicle can securely report accident-related transactions to its associated CH if it detects an accident on the road. The cluster head then securely transfers the received transactions to its associated RSU which in turn sends them secretly to the corresponding edge server. The edge server prepares a partial block containing the accident-related transactions, the Merkle tree root, and a digital signature. This incomplete block is forwarded to its associated cloud server in the blockchain center forming a complete block from the received partial block. At this point, all the cloud servers in the blockchain center participate in the block verification process through the PBFT consensus and if verified, the complete block containing the vehicle accident-related information is added to the blockchain center and made available for use by other vehicles for optimal route selection and better road-related decisions.

Using the Automated Validation of Internet Security Protocols and Applications (AVISPA) simulation tool, the proposed BCAS-VADN proves to be secure against multiple attacks including replay, man-in-the-middle attacks, impersonation and privileged-insider attacks, physical vehicle capture attack, and ephemeral secret leakage attack.

The authors in [125] introduced a scheme for securely authenticating the vehicles and the messages exchanged in the network using a public blockchain, called BCPPA. The scheme employs the Elliptic Curve Digital Signature Algorithm (ECDSA) but supports replacing it by an improved signature scheme to enable batch authentication. The BCPPA protocol consists of three phases: system initialization, message signing, and message verification. For improved security, the system initialization is performed by both the vehicles and the certificate authorities via the private-type derivation and the public-type derivation processes. The private-type derivation is performed by the vehicles to generate a root private key which is kept at the vehicle’s OBU to be used later to derive a fresh private key for each future communication. A corresponding public key is also generated by the vehicle and sent to the certificate authority which uses it to generate the new public key and certificate in the public-type derivation process. The public blockchain is used in this scheme to store the public certificates which are embedded into the transactions so that the vehicles can obtain the certificates from the blockchain instead of preloading all of them in the OBUs, which helps mitigate the storage burden of the vehicles. In the message signing phase, a vehicle that needs to broadcast a message to other vehicles in the network must sign the message by first executing the private-type derivation to obtain the current private key and then triggering the smart contract to obtain the transaction id that keeps the public certificate corresponding to the generated private key. The receiver then verifies whether the received message/signature pair is valid or not, if valid, the received traffic is accepted, and decisions can be made based on it.

Extensive simulation using Vanet-MobiSim and NS2 demonstrates that the proposed BCPPA can resist several attacks such as replay attack, impersonation attack, DDoS attack, man-in-the-middle attack, stolen verifier attack, side-channel attack, message modification attack, birthday collision attack, and hijacking attack. The authors claim good efficiency in terms of communication cost, time cost, average packet delay, and packet loss ratio.

The authors in [126] proposed a lightweight Decentralized Key Management Mechanism with Blockchain (DB-KMM) and bivariate polynomial. The network model involves three types of entities: Vehicle Service Provider (VSP), Blockchain Network (BN), and the vehicles. The VSP is responsible for deploying the BN and the smart contract, issuing the transaction data, registering, updating, and invalidating the vehicles’ public keys. The BN is constructed by the RSUs which are responsible for creating and mining the new blocks through the PoW consensus mechanism while providing public keys and services to the vehicles. The proposed DB-KMM is composed of six phases, namely, system setup, registration, authentication, key agreement, public key update, and public key revocation. In the system setup phase, the VSP derives the system parameters such as the ECDSA and the Elliptic Curve Integrated Encryption Scheme (ECIES) parameters, the bivariate function parameters as well as initializing the smart contract. Each vehicle that aims to join the VANETS network must register itself through the VSP which generates for it a pair of public and private keys while registering the public key in the smart contract. When a vehicle needs to communicate with other VANETS entities, it first mutually authenticates with the nearest RSU. Once the mutual authentication succeeds, a key exchange mechanism takes place between the vehicle and its corresponding RSU in order to agree on a shared session key for securing the subsequent communication.

The DB-KMM provides an automatic public key management including update and revocation via the smart contract. For the update process, when a vehicle sends a key update request containing its ID, old public key, and validity period to the VSP, the VSP triggers the smart contract to generate a new public/private key and a new validity period for the requesting vehicle. The VSP then forms a new transaction containing the vehicle ID, new public key, and validity period and sends it to the BN. The RSUs forming the blockchain network perform the consensus on the received transaction and if the mining succeeds, the updated transaction is added to the blockchain, and the new public key is transferred to the requesting vehicle. Lastly, when a malicious behavior is noticed on some vehicle, the VSP initiates a revocation transaction to the BN and triggers the smart contract to remove the vehicle’s identity and public key from the blockchain.

The performance of the proposed DB-KMM is tested in terms of the end-to-end packet latency and the packet loss ratio using OMNeT++ and Veins simulators and the results prove that it greatly improves the cost of public key management compared to the traditional PKI management. Further, security analysis shows that the scheme can resist DoS attack, public key tampering attack, internal attacks, as well as collusion attacks.

The authors in [127] extended the conventional blockchain by introducing two novel data structures called the Merkle Patricia Tree (MPT) and the Chronological Merkle Tree (CMT) and then proposed a Blockchain-based Privacy Preserving Authentication scheme (BPPA) based on this extended blockchain. The system model includes the following entities: certificate, certificate authority, Law Enforcement Authority (LEA), RSUs, and vehicles. The certificate includes the public key, the expiry date, the timestamp, and the encrypted mapping between the vehicle’s real identity and its certificates. The certificate authority issues two types of transactions: the issuance transaction which includes the issued certificate, the timestamp and the signatures of the trusted authorities, and the revocation transaction which contains the revoked certificate. The LEA is responsible for the registration of vehicles and monitoring their behavior. Additionally, it concatenates and organizes the transactions received from the certificate authority to generate a block and transfers the block to all the RSUs for verification. When a certificate issuance transaction or a certificate revocation transaction is broadcasted by the certificate authority, a leaf node is inserted into or removed from the MPT, respectively, and the root of the MPT is updated. The transaction and the corresponding root of MPT are recorded chronologically in the CMT.

The root of CMT is considered as the transaction root whereas the root of MPT is taken as the certificate root. The transaction root and the certificate root are then written immutably in the blockchain. The significance of this extended blockchain being developed is represented by two aspects. First, it provides a simplified authentication technique whether a certain certificate is in the MPT or not. Given the certificate root and a record containing the nodes along the path, the authenticator can compute a hash using the given record. If the hash value is equal to the certificate root in the blockchain, it is proven that the certificate exists in the MPT. Second, it provides transparency within the activities of the authorities by using transactions roots; since, if the transaction root is given, it can be verified when a certain certificate is issued or revoked. Conditional privacy preserving is provided by allowing each vehicle to utilize several certificates, while the mapping between the certificates and the real identities is encrypted by the LEA’s secret key and stored in the blockchain and can only be revealed by the LEA in case of malicious behavior. Security investigation proves that BPPA is resistant to forgery attack, man-in-the-middle attack, replay attack, identity revealing attack, and authority abuse attack. Further, experimental results demonstrate the efficiency of the scheme in terms of communication and computation costs, low latency, and high throughput.

The above-mentioned public blockchain-based authentication schemes can be found in Table 5 for easy reference.

### 4.3. Consortium Blockchain-Based Authentication Protocols

A consortium blockchain, also known as hybrid blockchain, is a combination of both private and public blockchain in which the read access can be open or restricted, and only a small group of nodes belonging to different organizations is responsible for making the consensus.

Since consortium blockchains incorporate the best of public and private blockchains, that is, a mixture of decentralization with good level of trust at the same time, it has been the most popular blockchain type being implemented by researchers for IoV authentication. In such way, the authentication can be performed with a high degree of trust since only a group of trusted entities perform the consensus of the authentication data blocks, while guaranteeing an efficient performance in terms of resource utilization and authentication time delay due to the semi-decentralized nature of consortium blockchains.

The authors in [128] optimized the Byzantine consensus algorithm by adopting time sequence and gossip protocol [129] to validate IoV information for correctness before adding them to the consortium blockchain. The use of gossip protocol, specifically the push–pull mode, enables faster data exchange among IoV nodes, as each two neighboring nodes can have the same information in one cycle. Two types of nodes are defined in the proposed scheme, i.e., Vehicle Communication Nodes (VCNs) and Roadside Communication Nodes (RCNs) in addition to the blockchain cloud platform used as storage for all IoV data. Due to their high computing and storage capabilities, RCNs are used as consensus-makers in the proposed Byzantine Consensus Algorithm with Time-sequence and Gossip protocol (BCA-TG). To ensure data integrity and authenticate the data sources, any data generated by a VCN or RCN must be agreed upon by more than half of the RCNs for the new block to be granted and linked to the blockchain.

In the BCA-TG protocol, each RCN has an Update Information (UI) vector containing the Local Information (LI) of all RCNs in the consensus network as elements. Initially, the UI of each RCN will have only its own LI while all the other LI elements corresponding to the other RCNs are set to null. For example, if 5 RCNs are used for consensus making, the UI of $RCN^{1}$ is initially: ${\mathrm{UI}}^{1}$ = {$LI^{1}$,0,0,0,0}. After which each RCN starts to communicate its UI vector with the neighbors in its view through the push–pull mode of gossip protocol until all RCNs have UIs with no null values. At this point, the element which forms more than half of the elements in the updated UI vector is considered the true information or the Consensus Information (CI) that will then be added to a new block and linked to the blockchain. The proposed scheme proves to have high fault tolerance since it can determine the CI even if the faulty or Byzantine nodes form 49% of the network. Moreover, their use of time sequence provides high scalability through its control over the entry and exit of nodes to/from the network and better convergence speed is achieved compared to the ordinary Byzantine consensus algorithm.

The authors in [130] handled the cross-datacenter authentication issue in Vehicular Fog Computing (VFC) environment by proposing BLA—a Blockchain-based Lightweight Anonymous authentication scheme. The system model consists of multiple regions; each region is managed by a Service Manager (SM) which is responsible for authenticating all OBUs and managing all Vehicular Fog Datacenters (VFDs) represented by the RSUs in its region. A Witness Peer (WP) exists in each region to write the authentication logs to the ledger maintained by the corresponding SM. The ledger is only accessible by the members of the consortium blockchain, such as the SMs, the WPs and the audit department which is assumed to be a fully trusted authority responsible for registering all the network entities underneath.

The RSUs within a region serve as access points to the vehicles in the IoV paradigm and also as fog computing units for providing real-time services to authorized OBUs. The proposed BLA protocol includes five phases: initialization, registration, authentication, consensus, and service-delivery phases. The initialization phase takes place only once via the initial setup of system parameters by the audit department. Then, the registration of SMs and OBUs is conducted by the audit department during the registration phase in which each registered entity is allocated a pair of private and public keys using Elliptic Curve Cryptography (ECC) and Diffie–Hellman (DH) cryptography mechanisms. During the authentication phase, each SM authenticates the OBUs within its region, and the authentication results are then passed to the subsequent phase where they are written to the ledger by the consensus makers, i.e., WPs, through PBFT consensus protocol. The way they addressed the cross-datacenter authentication is by allowing a flexible option to vehicles to choose whether to be reauthenticated or not upon entering a new VFD during the service-delivery phase.

The noninteractivity property that BLA has, in which a vehicle sends only one message to its SM for authentication or service requesting without the need for exchanging any acknowledgement messages between them, makes it lightweight in both communication and computation cost. In addition, the utilization of pseudonym preserves the privacy of vehicles and ensures anonymity. Security analysis proves that the proposed BLA protocol ensures most of the security aspects in IoV network, such as confidentiality, integrity, traceability, and non-repudiation. Moreover, extensive simulations are performed to measure the performance in terms of response time. The results obtained show that low time overhead is achieved which reflects the suitability of BLA algorithm for real-time VFS.

The authors in [131] improved the reliability of the authentication scheme proposed in [130] through the adoption of mutual authentication and key exchange mechanism. During the authentication phase, instead of just allowing one-way authentication in which only the SMs can check the authenticity of vehicles [130], a two-way authentication is enabled in [131] where a SM first authenticates the identity of a vehicle using the ECC then the vehicle authenticates the communicating SM in the same way. Upon successful mutual authentication, a session key is established and exchanged between the two parties for securing their future communication; which is a prerequisite for secure authentication protocol, unlike the work done in [130]. Moreover, the scheme guarantees the forward secrecy. The timestamps used for generating the authentication parameters prevent replay attacks. An extensive performance evaluation and comparison is conducted which reveals that the proposed solution outperforms the work in [130] in terms of communication and computational overheads.

The authors in [132] proposed a Blockchain-based Privacy preserving Authentication System (BPAS) for VANETs which supports password and biometric login-based authentication. BPAS relieves the overhead of having an online registration center during the authentication phase by providing a TA-independent authentication scheme in which vehicle’s authentication is handled by RSUs or other vehicles in the network. However, the TA is responsible for other system phases namely, system initialization, smart contract deployment, vehicle registration, and vehicle revocation phases. The scheme deploys a technique based on fuzzy extractor for biometrics extraction and an Attribute-Based Encryption (ABE) scheme to protect the privacy of users by encrypting their blinding identities to ensure that no other entity can decrypt them except for the blockchain managers.

During the system initialization phase, the TA initiates the system parameters including the ECC, the ABE, and the blockchain parameters. A smart contract is then deployed by the TA into the blockchain to automate the authentication process. Each vehicle then needs to be registered with the TA to obtain its secret authentication parameters during the registration phase. First, the owner of a vehicle must choose a physical identity, a password along with his/her biometrics which will be sent to the TA. The TA then calculates a corresponding blinding identity (AID) and a Vehicle Public Key (VPK) from the received parameters and uploads them as a unique tuple {AID, VPK} to the Vehicle Public Key Table (VPKT) in the smart contract. The AID is also sent to the vehicle’s OBU which is assumed to be tamper-proof. When a vehicle wishes to send data to nearby vehicles or RSUs, the vehicle’s owner provides the login information (i.e., password and biometrics) which are verified by the OBU. If correct, the OBU encrypts the vehicle’s AID using the ABE and sends it along with the message and a timestamp to the receiver (i.e., a vehicle or RSU), otherwise, the OBU rejects the message. The receiving entity then validates the freshness of the message through the timestamp. If valid, it initiates a transaction to the blockchain managers requesting for the associated vehicle public key. The blockchain managers then lookup the VPKT using the AID to obtain the corresponding VPK which is then transmitted to the transaction issuer to be verified. Upon successful validation, the authentication is completed, and the message is accepted.

BPAS also supports conditional traceability and vehicle’s revocation in case of detecting any malicious behavior by simply allowing the TA to delete the corresponding tuple in the VPKT. The proposed scheme is evaluated in terms of time cost and security features and is found to be resistant to replay attacks, impersonation attacks, DDoS, and password guessing attacks.

The authors in [133] proposed an Efficient Authentication Scheme over Blockchain for Fog computing-enabled IoV (EASBF). The authors consider three types of communication in their fog-enabled model, i.e., V2V, V2I, and V2R. Each fog area can provide different services to users and vehicles within its coverage range, and it includes one or more RSUs, one or more CAs, a single Blockchain Manager (BM), and a single Authentication Manager (AM). The CAs are trusted entities responsible for updating and managing the certificates issued for vehicles in their fog areas. The BMs are deployed to manage the blockchain and authenticate the OBUs whereas the AMs write the results of authentication into the blockchain (which together form the consortium blockchain). The PBFT is used for consensus.

The proposed scheme contains five phases: initialization, registration, mutual authentication and key exchange, consensus, and certificate update. A central secured entity, named TA, is responsible for initializing the system and the public parameters used for the cryptographic functions, as well as registering the OBUs and RSUs during the first two phases of EASBF. Then, mutual authentication and key exchange takes place between OBUs and BMs, and a session key is shared among them for subsequent interactions. Upon successful authentication and key exchange, the BM shares the results of authentication with all AMs, which store them into a new block and add it to the large public register under the PBFT consensus. During the consensus phase, one of the AMs acts as a “Speaker” which is responsible for initiating the consensus process while the rest serve as the congress members who participate in the voting scenario initiated by the Speaker. Finally, the certificate update phase which supports two scenarios: the ability to move from one fog area to another transparently without the need of re-authentication and certificate update upon a vehicle’s request. The Random Oracle Model (ROM) [134] and AVISPA tool [135] are deployed for formal security verification which proves the resistance of the proposed scheme against DDoS, replay, man-in-the-middle, identity theft, traffic analysis, masquerading, and session key disclosure attacks. Additionally, an extensive performance evaluation shows its efficiency in terms of computation, communication, and storage overheads.

The authors in [136] proposed an approach to address both anonymous authentication and efficient revocation of vehicles in VANETs through the use of pseudo-ids, blockchain, and revocation tags. The scheme defines three types of nodes, namely, a supervisory node which is the Traffic Department (TD), accounting and revocation nodes represented by the multiple TAs, and verification nodes which are the road-side units. The proposed scheme is composed of four phases: initialization, registration, mutual authentication, and expeditious revocation. The privacy of vehicles during authentication is preserved by using the pseudo-ids granted to them by the RSUs. When an RSU generates a pseudo-id for a vehicle during the registration phase, the RSU stores it into a Trusted Cloud Server (TCS) and transfers a pointer referring to the storage location of the pseudo-id to the corresponding TA. The TA then forms a transaction with the vehicle’s registration information including its public key certificate, the pointer to its pseudo-id stored in the TCS, and a unique Transaction id (TID) and uploads it to the blockchain. Using this TID, the identity of a vehicle can be later authenticated by viewing the corresponding records in the blockchain.

This arrangement in which the blockchain keeps only pointers to the pseudo-ids while the pseudo-ids themselves are kept in the unlimitedly huge TCS improves the scalability of the system. In addition, an illegal vehicle can be determined by checking whether its pseudo-id has a revocation tab instead of looking up the whole certificate revocation list which greatly reduces the computational overhead. Further, detailed security analysis proves that the proposed scheme can resist replay attacks and prevent single point of failure problem.

The authors in [137] addressed the interference issue caused by the continuous key updating in large-scale VANETS environments by proposing a blockchain-based framework for secure authentication and efficient group key updating in edge computing-enabled VANETs. They proposed a mutual V2R authentication scheme that employs certificateless cryptographic mechanisms in order to avoid the key escrow issue. The system model consists of a cloud layer which serves as a trusted authority, an edge layer represented by the distributed RSU clusters, and a user layer of OBUs.

The scheme is composed of two phases: offline registration phase and authentication phase in which each RSU authenticates a group of vehicles simultaneously as a batch which helps significantly in reducing the computation cost. Elliptic curve cryptography, one-way hash function, and bilinear pairing are utilized for generating secret key pairs and session keys during the offline registration phase and for securing communication during the authentication process. In the authentication phase, the shared session key of a vehicle is constructed independently which helps mitigate the interference in the regular V2R data exchange. In addition, an efficient group key management mechanism that employs the Chinese Remainder Theorem (CRT) [138] is suggested by the authors for reliable and secure V2V communication. A consortium blockchain is deployed during the dynamic group key updating process to record the identity of the participating vehicles in order to provide traceability of vehicle’s historical data when needed. Formal security analysis proves the resiliency of the proposed scheme to chosen message attacks and replay attacks. Low storage, communication, and computation overheads are also recorded through deep performance evaluation.

The authors in [139] designed a novel data structure based on the idea of the Unspent Transaction Output (UTXO) adopted in Bitcoin in combination with a group of online operations, namely, issue, transfer, query, and revocation. The system framework consists of two layers: the entity layer which is mainly the vehicles and RSUs that need authentication service and the trust layer represented by the TAs and the consortium blockchain. Each TA is responsible for managing a dedicated group of entities which all together form an organization. When creating a new block, a sufficient number of organizations must sign it to be accepted to the consortium blockchain. However, the authors developed the use of tokens in the UTXO to serve as a one-time guarantee for the authenticity of an entity instead of using incentives as in Bitcoin. Once an entity receives a token from another entity in the public ledger, this not only means an authentication request being issued, but also proves or guarantees the authenticity of the dedicated sender.

The UTXO data structure is formed by three key items, namely, basic, in, and out items. The basic item includes the transaction id, the operation name, the timestamp, and the signature of the requester to prove its ownership. The in item represents the information of the sending entity while the out item includes information related to the receiving entity. The authors define several operations that are as follows. The Issue operation is used by trusted authorities to generate new tokens for the entities, which can take place only upon two circumstances: the initial enrollment of a vehicle and periodically generated for well-behaved entities. Similarly, the Query operation can be used to retrieve the UTXO of a transaction on the blockchain through transaction id in order to check the trustworthiness of a sending entity. The Revoke operation is the key operation introduced by the authors for revocation management instead of the conventional certificate revocation list that implies extra storage and computation overheads. However, the main operation used for authentication purposes is the Transfer operation and the procedure is as follows: when an entity $E_{i}$ sends an authentication request to Entity $E_{j}$, Entity $E_{j}$ extracts the unique transaction id from the request message and uses it to query and retrieve the transaction UTXO from the blockchain. Then, the identities of the sending and receiving entities in the retrieved UTXO are compared against the ones received in the authentication request to check their legitimacy. If confirmed, the existence of the UTXO should be verified in the receiving entity’s ($E_{j}$‘s) local database to check for token reuse. If the UTXO does not exist, the authentication is successful and it is recorded immediately in the database for future authentication references, otherwise, the authentication is rejected. In addition to resisting replay attacks (due to the use of timestamps and one-time tokens), the scheme prevents man-in-the-middle attack, identity revealing attack, as well as authority abuse attack.

The above-mentioned consortium blockchain-based authentication schemes can be found in Table 6 for the ready reference.

## 5. Discussion

In Table 4, Table 5 and Table 6, we provide a summary of the blockchain-based authentication papers surveyed in Section 5. The table highlights the main properties of each scheme which are represented in the blockchain and cryptographic techniques used, the network model designed, the tools used for security verification and performance evaluation, the features, and limitations (if any), and whether each scheme supports a privacy preservation option for user’s identity during the authentication process or not. From the table, we can conclude the following:

1. The choice of the consensus mechanism used by the blockchain depends on many factors such as the amount of power consumption the system can afford, the percentage of faulty nodes the network can tolerate, and others. However, the size of the IoV network is also an important factor to be considered. For example, although the PBFT consensus is lightweight in power consumption and has a good fault-tolerance of approximately 33%, it does not support high network scalability; thus, it is chosen only by the small-scale IoV platforms such as in [128], or by fog-based IoV networks where the whole network can be large but the consensus is performed in fog-area-basis such as in [123,130,131,133]. On the other hand, PoW has been chosen by the large-scale IoV environments [119,122,125,126] due to its high scalability and high fault tolerance, despite its large power consumption. In general, we can say that these two consensus mechanisms are the most commonly used by the blockchains when applied for IoV and VANETs authentication.
2. Some of the papers only considered a V2V model to investigate their authentication schemes such as [120], while most of them have considered an integrated network model (V2V and V2I). Due to the ad-hoc and mobile nature of V2V communication, the authentication is more challenging in it.
3. Most of the papers support anonymous authentication schemes, while some of them provide pure authentication without supporting such privacy preservation option as in [111,120,126,128].
4. In terms of performance analysis, the use of blockchain for authentication in IoV and VANETS networks introduces the need to include additional evaluation measures that reflect the efficiency of the blockchain itself such as the throughput which refers here to the number of transactions that can be verified and added to the blockchain per second [117,119,127,139], the transactions latency [119,122,127], and the blockchain storage overhead [123,126,133,136,137], beside other measures that are used commonly to evaluate the conventional cryptographic-based authentication schemes including the average packet delay, the average packet loss ratio, the communication cost, and the computational cost.
5. Regarding the security analysis, the different authentication schemes have shown uneven levels of resistance against the various security attacks introduced in the previous sections.

To conclude, a comprehensive view of the recent blockchain-assisted authentication schemes in IoV and VANETs was provided in this survey while highlighting the main differences between them in terms of security performance and operational performance. This helps in identifying the research gaps and creates a map that guides the future researchers in this area of IoV blockchain-based authentication. Although the adoption of blockchain for IoV authentication has brought many benefits such as the increased time-efficiency due to the decentralization of the authentication scheme among the distributed blockchain servers, and the more secure authentication reflected by the high resistance against different security attacks, a lot of work still must be done. This area needs more research and development to design new IoV-specific consensus mechanisms that can provide a more efficient balance between scalability and power consumption by consuming less power, supporting highly scalable IoV networks while maintaining the same good level of security represented by the high fault tolerance.

## 6. Future Research Directions and Open Challenges

In this section, a number of research challenges in IoV security that need to be addressed as well as some potential research directions to be explored are suggested.

### 6.1. Efficient Design of Blockchain-Assisted IoV Authentication

Despite the many advantages that blockchain technology has brought to the field of IoV authentication represented by a decentralized, autonomous, fault-tolerant, and more secured authentication protocols which are all properties that are required by any efficient authentication process, many challenges also arise with blockchains when being employed for authentication in environments with high performance expectations and critical security requirements such as the IoV. Most of these challenges are associated with the consensus mechanisms used by the blockchain, as different consensus protocols show uneven levels of support for the various IoV requirements such as scalability, fault tolerance, power consumption, as well as real-time response.

(1) Scalability: The scalability of a consensus mechanism depends on its way of reaching the consensus, whether it is Proof-based or Vote-based approach. Since Proof-based consensus protocols such as PoW and PoS do not require all the network entities to submit their individual decisions on the information to be verified, their scalability is not affected with more nodes being added to the network as at the end of the day they allocate the role of announcing the conclusions of all the participating nodes about the information to a single node. Thus, they can be suitable for large-scale networks such as the IoV environment. However, the huge amount of power consumed by these Proof-based consensus approaches undermines their efficiency and their suitability for all IoV environments especially the resource constrained ones. On the other hand, the Vote-based blockchain consensus making approaches such as the PBFT consensus mechanism exhibit negligible power consumption, yet their scalability is restricted to small-scale networks with limited number of nodes since all the nodes in the network are engaged in transaction verification and should submit their individual votes in order to reach consensus. For such a trade-off that is faced by the existing consensus mechanisms when employing blockchain for IoV, the challenge is represented in how to tune and improve these mechanisms in the future to be able to support efficient authentication in the highly scalable resource constrained IoV environments.
(2) Fault tolerance: the different blockchain consensus mechanisms have uneven capabilities in terms of how many faulty nodes each can tolerate while still being able to ensure the integrity of the participants and data in the network. In such wide and publicly opened environments as IoV where a variety of attacks can be encountered in which attackers impersonate the authentic nodes, high fault-tolerant consensus protocols should be adopted to ensure that the integrity of the communicated data can still be guaranteed even if the authenticity of a considerable percentage of participants is compromised. Some existing consensus protocols such as PoW can offer the high fault tolerance required for IoV environments; however, as discussed before it exhibits a large power consumption which can be an issue when used for authentication in some resource constrained IoV platforms. Thus, the need appears for developing new consensus making approaches that can guarantee high fault tolerance for the vulnerable IoV arrangements while maintaining an acceptable level of power consumption. Alternatively, mitigating the power consumption effect of the already existing blockchain consensus protocols by finding other solutions such as the charge-as-go solution represented by providing mobile charging units to charge the vehicles’ batteries as they move to be able to tolerate the high power consumed during the authentication is a challenging proposal to be explored in the future.
(3) Communication mode: Due to the two types of communication used by the different consensus mechanisms which are synchronous and asynchronous communication modes, different time responses are expected. In the asynchronous mode, the sending entity does not wait for acknowledgement from the receiving node on a previous request, instead, it directly proceeds with the following communication steps. The consensus protocols that use this mode of communication such as Proof of Work (PoW) and Proof of Time (PoT) could be thought of as perfectly suitable to be used for authentication in the different IoV applications which are mostly time-critical applications in which even a small delay of milli seconds is not forgivable. However, when considering some availability issues scenarios where the receiving entity can be temporarily out of network, waiting for an acknowledgement from the receiving entity before proceeding on with further interaction would save a lot of time and bandwidth that were used for such useless communication. Therefore, developing novel consensus making protocols that combine synchronous and asynchronous modes into one hybrid mode that incorporates the advantages of both communication modes, for example, by setting some thresholds for the number of acknowledgments to be received before proceeding the rest of communication asynchronously, or by using timeouts. However, such proposed solution is in turn full of challenges, since this hybrid mode of operation needs to be repeated periodically and regulated with other thresholds, that is, the synchronous mode should be injected once after every consecutive asynchronous interaction to check periodically that the receiving entity can be reached. This arrangement imposes an extra overhead associated with designing the optimal thresholds, monitoring, and managing the different time windows and timeouts. Thus, the feasibility of developing such complicated hybrid consensus protocols to be used for IoV authentication while maintaining a relatively low operational cost is another future challenge to be investigated.

Thus, to design an efficient authentication protocol for IoV based on blockchain that maintains a good performance balance while highly considering all the above-mentioned factors is a great challenge that should be addressed by future researchers. In the way to achieve this, serious efforts should be dedicated to developing all-in-one IoV-specific consensus mechanisms that can meet all the requirements of IoV applications including high scalability, high fault tolerance, real-time response with low energy consumption. Beside these factors, another important challenge in this area to consider is how to achieve the optimal assignment of the various blockchain-related tasks such as blocks creation, validation, and consensus making to the different IoV nodes based on their computing capabilities and the energy consumption requirements of the adopted consensus protocols in order to achieve an efficient authentication process. In [119] for instance, blocks creation is assigned to the infrastructure units through PoW due to their high computing power, whereas blocks verification is assigned to the vehicles through PBFT consensus due to their relatively low computing capabilities. However, more technical details and conditions need to be fully explored in upcoming research studies.

### 6.2. Employing Blockchain for other IoV Security Requirements

In this survey paper, we targeted the adoption of blockchain in IoV for the authentication security requirement. However, we believe that blockchain can be of great benefits for other IoV security requirements such as data integrity, secure routing, and availability which can all be explored and discussed as part of the future research directions.

(1) Data Integrity: Blockchain can be adopted by IoV environments to ensure that the data sent and received between two communicating IoV entities are identical. In other words, to make sure that no unauthorized modifications on the data take place during transmission. For instance, when a sender aims to send some data to another IoV node, it can send a copy of the data to the publicly accessible blockchain as well. At the other end, when the receiving entity receives the data, it can compare it with the copy stored in the immutable blockchain, if matched that means no manipulation was performed and data integrity is guaranteed. Otherwise, the received data cannot be trusted. The use of blockchains for ensuring data integrity in IoV may become even more important to explore when considering using them for big data analytics and data mining which require the data to have high level of accuracy to result in developing accurate and efficient decision making and AI-based applications.
(2) Secure Routing: Blockchain technology can be used for guaranteeing a secure routing in IoV environments via different arrangements. An example could be maintaining a list of all possible legal next-hop nodes in the public ledger which can be accessed by all IoV entities. This list should be firstly developed by gradually adding the ids of the nodes that were successfully registered and authorized by the network. This means that any malicious unauthorized node will not be valid in this routing list. In this way, when an entity receives a packet that was routed through at least one illegal forwarder that does not exist in the routing list kept in the blockchain, this means the routing processes have been compromised and some illegal entity had access to the transmitted packet.
(3) Availability: A feasible suggestion that could be investigated is the integration of blockchain with some physical identification modules in an attempt to prevent some availability attacks and threats. That is, obligating each single service request to include the physical identity of the request originator, e.g., MAC address of the NIC of the user’s mobile device or the vehicle’s hardware id assigned by the vehicle manufacturer. Then, upon receiving the request by an infrastructure node, i.e., a RSU, it should extract the physical identity of the requester and store it as a record in the blockchain while keeping track of a counter that counts the number of successive requests and a timer to monitor the time intervals between these successive requests. In such arrangement, availability attacks specifically the denial of service (DoS) attack that originates from a single physical entity with different logical identities, i.e., IP addresses, can be efficiently detected and thus blocked by any IoV entity through monitoring the public ledger records and noticing any extraordinary behavior of rapid successive requests originated from the same physical id.

The above-mentioned examples are a few humble suggestions for using blockchain in the context of the different IoV security requirements. However, there is a strong belief that blockchain capabilities can cover more aspects and offer a variety of solutions regarding these IoV security concerns, yet to be explored.

### 6.3. Cloud Scalability, Security, and Privacy

Since IoV paradigm is based on big data and high-performance computing, cloud computing infrastructures constitute the most important building blocks for providing data storage. Even in blockchain-assisted IoV platforms, the cloud servers (CS) cluster constitutes the blockchain network.

Thus, developing the cloud technologies is a critical aspect to guarantee the security and the privacy of the blockchain-based authentication system in vehicular networks. Cloud-related security and privacy issues might be encountered during the process of transmitting vehicles’ data to/from the cloud or while being stored in the different cloud servers. This imposes different security challenges in both processes which should be addressed in the future to guarantee a high level of efficiency for the blockchain-based cloud-assisted IoV authentication systems.

Data transmission challenges: Transmitting data back and forth between the cloud servers, the vehicles, and the other IoV entities via an open wireless network may impose high risks on the security of the blockchain-based authentication system and the privacy of the users. Some schemes attempt to mitigate the security risk by adopting wired communication wherever possible, i.e., between the cloud servers and the intermediate nodes. However, this arrangement is not practical nor feasible for all IoV-based scenarios. Moreover, this can only mitigate the risk at the stationary end of the system while the mobile part, i.e., the V2I communication is still exposed to different security threats due to its wireless nature. Thus, the existing wireless communication protocols should be well investigated and developed to insure private and secure exchange of users’ authentication data between the different parts of the IoV-based system.

Data storage challenges: Having users’ sensitive data such as the authentication data including users’ real identities, public keys and certificates stored in a common-access platform such as the cloud is another threat to consider. The privacy of users is threatened to be violated if no proper encoding schemes exist within the cloud. Thus, the need for developing efficient encryption algorithms that can be adopted in both data storage and transmission to protect the privacy and security of the whole IoV cloud-based paradigm while ensuring full compatibility with the rapidly evolving and heterogenous cloud technologies is another important challenge which we hope to be tackled in the future.

## 7. Conclusions

In this survey, security aspects of the emerging vehicular technology, IoV, and the preceding VANETS which have made an evolution in the intelligent transportation systems were discussed. The power of the emerged blockchain technology in general and specifically in IoV was also highlighted. Moreover, different security requirements, challenges, and potential security attacks and threats in vehicular networks were presented. After that, more focus was dedicated to discussing a wide range of recent blockchain-based authentication techniques in IoV and VANETs environments and a comprehensive comparison between them was then provided. At last, some possible security challenges and research directions in IoV and VANETs that need to be addressed in the future were highlighted. In this paper, we focused only on the conceptual comparison between the surveyed blockchain-based IoV authentication schemes, i.e., in terms of the different techniques, network architectures, and evaluation tools used, as well as features and limitations. However, we believe that including more quantitative measures in comparisons is a direction that can be considered and improved in future surveys.

## Figures and Tables

**Table 1:** Comparison of recent surveys on authentication in IoV and VANETs networks.

Ref.	Year	Target Area	VANETs to IoV Transition	Security Attacks and/or Requirements	Blockchain-Based Authentication	Features
[47]	2017	IoT	X	√	X	Discusses symmetric and asymmetric cryptographic-based authentication protocols. Covers authentication protocols in a wide range of IoT environments, namely, IoV, IoS, IoE, and M2M. Presents threat models, countermeasures and formal security verification techniques used by the surveyed papers.
[48]	2017	VANETs	X	√	X	Surveys a range of authentication schemes that are based on cryptography, digital signature, and message verification. Provides a performance comparison in terms of communication and computation overheads.
[49]	2019	IoT	X	√	X	Provides a multi-criteria classification for the surveyed authentication schemes which includes authentication factor, procedure, and architecture, IoT layer, use of tokens and use of hardware. Presents different security requirements and issues faced by each IoT layer.
[50]	2019	VANETs	X	√	X	Discusses authentication and privacy schemes in VANETs while providing a good taxonomy based on the privacy preserving technique used. Presents the security of each scheme in terms of security requirements and their corresponding attacks. Shows performance efficiency w.r.t computational cost and communicational cost for each scheme.
[51]	2020	VANETs	X	X	X	Addresses authentication, privacy, and secure message dissemination in VANETs. Proposes multi-categorization based on the tools and techniques used in the surveyed papers.
[52]	2020	IoV	√	√	X	Provides a good taxonomy of various security protocols in IoV. Surveys authentication protocols in IoV. Discusses security aspects: threats and attacks in IoV. Provides a performance comparison in terms of communication and computation overheads.
[53]	2020	IoV	√	X	√	Provides a comprehensive comparison of the blockchain-based applications in vehicular networks. Analyzes the requirements of the blockchain-based applications in vehicular networks. Discusses a range of challenges related to the integration of blockchain within vehicular networks.
[54]	2021	IoV	√	X	X	Provides seven different aspects for combining the blockchain technology with IoV while briefly surveying some schemes for each of these aspects. Overviews some research directions in the field of blockchain-enabled IoV.
[55]	2021	IoV	X	√	√	Provides a detailed review on various existing blockchain techniques for IoV security. Provides a good categorization for the existing blockchain-based IoV security methods. Presents a clear analysis for the surveyed blockchain-based IoV security schemes in terms of techniques, tools, and performance metrics. Discusses a couple of future research aspects.
Our survey	2021	VANETs and IoV	√	√	√	Covers the specific area of VANETs and IoV, which provides a more focused reference for researchers in the field of IoV, meanwhile a more comprehensive reference in vehicular technology and ITS. Highlights the efficiency of blockchain in IoV by discussing blockchain-based authentication schemes. Provides a clear taxonomy in terms of the type of blockchain used for authentication. Presents a detailed comparison between the surveyed papers in terms of techniques used, attacks counteracted, network models, and evaluation tools. Discusses whether each authentication scheme supports privacy preservation of user identity or not. Focuses on the attacks on authentication, their targeted OSI layers, and possible remedies.

**Table 2:** Possible security attacks in IoV and VANETs categorized by security requirements.

Attack Targets	Description	Examples	Attack Type
Availability	Attacks that affect the IoV system’s availability normally use techniques that can make the bandwidth and transmission power of the IoV system unusable by occupying its maximum resource capacity.	- DoS attack	Active
		- DDoS attack	Active
		- Channel interference attack	Active
Authentication	Attacks that fake the identity of the original sender and act on its behalf, which could be used to spread harmful information in the network.	- Sybil attack	Active
		- Masquerading/Impersonation attack	Active
		- Wormhole/tunnelling attack	Passive/Active
		- GPS spoofing attack	Active
		- Replay attack	Active
Data Integrity	Attacks that tamper with the original message content to badly affect the decisions of the receiving entity which could threaten the overall system.	- Message manipulation attack	Active
		- Malware attack	Active
Confidentiality	Attacks that compromise the privacy of data through receiving unauthorized copies of the messages being transmitted between the legitimate sending and receiving entities by an illegal third party.	- Man-in-the-middle attack	Passive/Active
		- Eavesdropping attack	Passive
		- Message holding attack	Active
Routing	Attacks that manipulate the original route of a packet by injecting some malicious recipients in the middle to eavesdrop or even sometimes alter the message before further forwarding it towards its targeted destination.	- Eavesdropping attack	Passive
		- Denial of service attack	Active
		- Masquerading attack	Active
		- Route modification attack	Active

**Table 3:** Possible attacks on authentication, their targeted OSI layers, and remedies.

Attack	Targeted OSI Layer	Possible Solutions	References
Sybil attack	Network layer	Group signatures, session key certificates, event based reputation system, footprint	[100,101,102,103]
GPS deception attack	Physical layer	Dead reckoning, signature-based mechanisms	[104,105]
Masquerading/Impersonation attack	Multi-layer (Physical, Data link, Network, Transport, and Application layers)	Digital certificates, identity-based cryptography	[106,107]
Wormhole attack	Network layer	Geographical leashes	[108,109]
Replay attack	Network layer	Timestamps	[110]

**Table 4:** A comparative study of the private blockchain-based authentication schemes in IoV and VANETs.

Ref.	Technique	Attack Counteracted	Network Model and Entities (Other than Blockchain)	Evaluation	Metrics	Features and Limitations	Privacy Preservation of Vehicle’s Identity?
[111]	- Rayleigh consensus mechanism - Intelligent contract - PKI based on password accumulator	- Not specified	- V2I - vehicles - RSUs - Trusted centre (cloud service provider)	- Veins simulation tool (OMNeT++ and SUMO)	- Computational cost (16.5 ms) - Communication cost (50 KB)	- Provides an efficient key distribution mechanism based on crypto-accumulator - Provides mutual authentication and key exchange - Large packet loss in the vehicle registration and key distribution process - No security analysis	No
[112]	- -Delegated Proof-of-Stack (DPoS) consensus mechanism - Smart contract	- Eavesdropping - Replay - VN capture and VN impersonation - RSU and TA spoofing - Jamming - Session fixation - Wrong password login/update	- V2I - Vehicle nodes - RSUs - Trusted authorities - Data centre	- ProVerif tool	- Computational cost (0.434 ms) - Communication cost (4416 bits)	- Supports an efficient cross-TA authentication - Improves the authentication efficiency through offloading part of the computational load to the RSUs to reduce the TAs’ resource bottleneck - Lightweight in computation - Does not consider the design of the communication protocol between the TAs and the DC and relies on assuming a secure channel between them, which is so idealized and lacks rationality	Yes
[115]	- Smart contract - Asymmetric key cryptography	- Identity spoofing	- V2I - Vehicles - RSUs - A single registration server - Multiple service providers	- Software implementation using Solidity language on Remix platform	- Not specified	- Supports an optional traceability feature. - Supports conflict-free access-log maintenance. - No performance evaluation	Yes
[117]	- Proof-of-Authority consensus mechanism - Elliptic curve discrete logarithm problem - Elliptic Curve Digital - Signature Algorithm	- Impersonation	- V2I - OBUs - RSUs - Certificate authority - Revocation authority	- OMNeT++ (Objective Modular Network Testbed in - C++) - Veins simulation tool	- Communication delay (45 ms for up to 5 vehicles) - -Throughput (45 bits/s for up to 5 vehicles) - Packet delivery ratio (90% up to 35 vehicles, then drops with increasing traffic from 35 to 50 vehicles)	- Provides mutual authentication with reduced dependency on the CA thus reducing the overall communication overhead - Ensures data confidentiality, integrity, and non-repudiation - Supports fast revocation of vehicles without extra overhead - No single point of failure - Does not fully explore the characteristics of the blockchain technology such as the use of smart contracts	Yes
[119]	- Proof of Work (PoW) consensus mechanism - Practical Byzantine fault tolerance (PBFT) consensus mechanism - Elliptic curve cryptography (ECC) - AES-256	- Internal impersonation	- V2I - Vehicles - RSUs - A root trusted authority - Local trusted authorities	- Simulation model implemented with Python	- Total consensus delay (increases linearly from 2 s at 25 vehicles to 10 s at 150 vehicles) - Transactions per second (decreases exponentially from 50 TPS at 25 vehicles to 10 TPS at 150 vehicles)	- Guarantees message integrity - Ensures non-repudiation and traceability - Prevents single point of failure - Large delay in consensus making	Yes

**Table 5:** A comparative study of the public blockchain-based authentication schemes in IoV and VANETs.

Ref.	Technique	Attack Counteracted	Network Model and Entities (Other than Blockchain)	Evaluation	Metrics	Features and Limitations	Privacy Preservation of Vehicle’s Identity?
[120]	Link fingerprinting	Replay	- V2V - 2 vehicles - (With MICAz mote mounted on each) - Fusion centre (Cloud server)	- Hardware implementation using MICAz motes - Simulation using MATLAB R2020a	- Pearson Correlation Coefficient - Computational time (s)	- Provides real-time adversary detection. - Lightweight in computation - Lack of security evaluation	No
[122]	- Dynamic Proof-of-Work (dPoW) consensus mechanism - Smart contract - PKI - Physical unclonable functions	- Cloning - Impersonation - Data tampering	- V2I - Vehicles - RSUs - Miners - Cloud storage - PUFs	- Software implementation (using Solidity for smart contract and Python for dPoW) - Simulation using SUMO and NS3	- Communication overhead (bytes) - Latency (s) expressed in 2 measures: - 1—Authentication delay at RSU - 2—Time-to-Finality - MAC and physical layers bytes overhead (%)	- Supports scalability according to the incoming traffic - Satisfies all the four-way trade-off properties (scalability, decentralization, low latency, and security guarantee) - Provides physical protection through the PUFs	Yes
[123]	- PBFT consensus mechanism - Symmetric key: Advanced encryption standard - Asymmetric key: Elliptic curve discrete logarithm problem - Bilinear pairing	- Replay - Stolen verifier - Data poisoning - Man-in-the-middle - Privileged-insider - Vehicle and RSU impersonation - Ephemeral secret leakage	- V2I - Vehicles - RSUs - Trusted authorities - Fog servers - Cloud servers	- Automated Validation of Internet Security Protocols and Applications, simulation tool for formal security analysis - MIRACL: library for measuring the execution time of the different used cryptographic techniques	- Storage overhead (2720 bits) - Communication cost - (Single and batch verification) - group key management - Computation cost - (V2V single authentication and V2RSU batch authentication)	- The genuineness and authenticity of blockchain data supports the use of big data analytics to machine learning and AI applications - Supports batch authentication which saves time and reduces the computational overhead - Does not provide practical implementation on the claimed support for big data analytics, AI, and ML	Yes
[124]	- PBFT consensus mechanism - Elliptic curve discrete logarithm problem (ECDLP) - One-way hash function - ECDSA	- Replay - Man-in-the-middle - Impersonation - Privileged-Insider - Physical vehicles capture - Ephemeral secret leakage	- V2I - Vehicles - RSUs - Registration authority - Edge servers - Cloud servers	- Automated Validation of Internet Security Protocols and Applications (AVISPA) simulation tool for formal security verification	- Communication cost (2400 bits) - -Computation cost (227.6 ms)	- Enables vehicle accident detection and notification - Supports mutual authentication and key agreement - Does not discuss the blockchain-related evaluation measures, i.e., the throughput (TPS) and the blockchain’s storage overhead	Yes
[125]	- PoW consensus mechanism - Smart contract - ECDSA	- Hijacking - Impersonation - Message modification - DDoS - Replay - Man-in-the-Middle - Stolen verifier table - Side-channel	- V2V - Vehicles - RSUs - Certificate authorities	- Vanet-MobiSim simulation tool - NS2 simulation tool	- Time cost (0.017974 s). - Communication cost (264 byte) - Average packet delay (APD in s) - Packet loss ratio (PLR in %)	- Supports batch verification - Does not consider the real-world factors in security and performance evaluation	Yes
[126]	- PoW consensus mechanism - Smart contract - Bivariate polynomial - ECDSA - ECIES	- Internal - Public key tampering - DoS - Collusion	- V2I - Vehicles - Blockchain network: consists of the RSUs - Vehicle service provider	- OMNeT++ event simulator - Veins network simulator - OMNeT++	- Computation overhead (ms) - Communication overhead (657 bytes) - Storage overhead (114.4 MB) - Average end-to-end packet latency (ms) - Average packet loss ratio	- Supports mutual authentication - The authentication process is lightweight - Does not support anonymity during authentication	No
[127]	- Elliptic curve cryptography - ECDSA - Secure hashing algorithm - Advanced encryption standard	- Forgery - Man-in-the-middle - Replay - Identity revealing - Authority abuse	- V2I - Vehicles - RSUs - Certificate authority - Semi-TAs - Law enforcement authority	- Testbed (2 laptops as RSUs and 6 laptops as vehicles connected through 1 Gb/s switch) + Software implementation using Python	- Transaction throughput (transactions/s) - Transaction latency (ms) - Time consumption (ms) - Communication overhead (KB)	- Supports a conditional privacy - Ensures integrity and non-repudiation - The scheme is evaluated on a small-scale IoV platform (only 2 RSUs and 6 vehicles) which is not enough to prove its efficiency in real-world scenarios	Yes

**Table 6:** A comparative study of the consortium blockchain-based authentication schemes in IoV and VANETs.

Ref.	Technique	Attack Counteracted	Network Model and Entities (Other than Blockchain)	Evaluation	Metrics	Features and Limitations	Privacy Preservation of Vehicle’s Identity?
[128]	- Byzantine consensus mechanism - Gossip protocol and time sequence	Byzantine/faulty nodes attack	- V2I - Several VCNs - 5 RCNs - Data storage (multiple servers)	Mathematical modeling	Simple data comparisons	- Fast convergence speed. - Good Byzantine fault tolerance. - Good control over the entry/exit of multiple nodes to/from the network. - Lack of security analysis and performance evaluation.	No
[130]	- PBFT consensus mechanism - Asymmetric key crypto - Elliptic curve discrete logarithm problem	Impersonation	- V2I - OBUs - RSUs - Service managers - Witness peers - Audit department	- Simulation - Hardware components: OBU, RSU and SM represented by 3 PCs to measure the transmission delay	Time overhead (ms)	- Ensures Confidentiality and integrity of data. - Ensures traceability and non-repudiation of misbehaving vehicles. - Provides flexible cross-datacenter authentication. - Does not support mutual authentication. - Does not provide formal security analysis.	Yes
[131]	- PBFT consensus mechanism - Asymmetric key cryptography - Elliptic curve cryptography	- Impersonation - Replay	- V2I - OBUs - RSUs - Service managers - Witness peers - Audit department	- Automated Validation of Internet Security Protocols and Applications (AVISPA) simulation tool for formal security verification	- Computational overhead (ms) - Communication overhead	- Ensures confidentiality and integrity of data. - Ensures non-repudiation. - Supports non-interactivity, thus lightweight. - Supports forward secrecy. - Supports mutual authentication and key exchange. - Supports cross-datacenter authentication. - Does not discuss the details of the security analysis, i.e., threat model, assumptions, etc.	Yes
[132]	- PBFT consensus mechanism - Smart contract - Attribute-based Encryption - Elliptic curve cryptography - Fuzzy extractor	- Replay - Vehicle impersonation - Offline password guessing - DDoS	- V2I - The upper layer: Trusted authority (TA) - The bottom layer: RSUs and vehicles	- Software implementation using relic library for the time cost of the cryptographic operations - JavaScript on Hyperledger Fabric platform for the smart contract	Time overhead (5.693 s)	- Supports traceability and dynamic revocation of mis-behaving vehicles. - Does not evaluate the scheme in terms of communication overhead and storage overhead.	Yes
[133]	- PBFT consensus mechanism - Elliptic curve cryptography - One-way hash function	- DDoS - Replay - Man-in-the-middle - Identity theft - Traffic analysis - Masquerading - Session key disclosure	- V2V, V2I and V2R - Vehicles (OBUs) - RSUs - Trusted authority - Certification authority - Authentication manager - -Blockchain manager - Fog area	- Automated Validation of Internet Security Protocols and Applications (AVISPA) simulation tool and Random Oracle Model (ROM) - C++ software implementation under Visual Studio using Crypto++ library	- Computation overhead (91.04 ms) - Communication overhead (24 tokens) - Storage overhead (186 bytes)	- Guarantees the confidentiality and the integrity of data. - Guarantees traceability and non-repudiation. - Ensures perfect forward secrecy. - Supports non-interactivity. - Provides mutual authentication and key exchange.	Yes
[136]	Elliptic curve cryptography	Replay attacks	- V2I - OBUs - RSUs - TAs - Trusted cloud server - Traffic department	Not specified	- Time consumption (ms) - Storage capacity	- Ensures the confidentiality of data. - No single point of failure. - Reduces the computational overhead associated with vehicles’ revocation. - Supports system scalability. - Insufficient evaluation.	Yes
[137]	- Elliptic curve crypto - Bilinear pairing - One-way hash	- Replay - Chosen message attack (CMA)	- V2V and V2R - Cloud server - RSUs - OBUs	- Their customized simulation	- Computation cost (ms) - Storage overhead (bits) - Communication cost (rounds)	- Provides efficient key updating that avoids the interference in V2R data exchange. - Provides efficient group key distribution scheme. - Conditional anonymity - Unforgeability - Formal security analysis is required.	Yes
[139]	- Smart contract - Public–private key pair cryptography	- Replay - Man-in-the-middle - Identity revealing - Authority abuse	- V2I - Vehicles - RSUs - Trusted authorities	- Testbed + Software implementation (a LAN of 4 nodes with a smart contract deployed over them)	- Time cost (ms) - Throughput (transactions per second)	- Reduced storage and computation overheads associated with the revocation process due to the discard of certificate revocation lists (CRLs). - Does not evaluate the scheme in terms of communication overhead and storage overhead.	Yes

## Data Availability

Not Applicable.
